# The Impact of COVID-19 on Racialised Minority Populations: A Systematic Review of Experiences and Perspectives

**DOI:** 10.3390/ijerph22121767

**Published:** 2025-11-21

**Authors:** Toni Wright, Raymond Smith, Rajeeb Kumar Sah, Clare Keys, Harshad Keval, Chisa Onyejekwe

**Affiliations:** 1 School of Nursing, Midwifery, Allied and Public Health, Canterbury Christ Church University, North Holmes Road, Canterbury, Kent CT1 1QU, UK; raymond.smith@canterbury.ac.uk; 2 School of Human and Health Sciences, University of Huddersfield, Queensgate, Huddersfield HD1 3DH, UK; r.k.sah@hud.ac.uk; 3 Kent and Medway Mental Health NHS Trust, Research and Innovation Centre, Beech House, Hermitage Lane, Maidstone ME16 9PH, UK; 4 School of Applied Sciences, Edinburgh Napier University, Sighthill Campus, Sighthill Court, Edinburgh EH11 4BN, UK; h.keval@napier.ac.uk; 5 Bristol Law School, University of the West of England, Frenchay Campus, Coldharbour Lane, Bristol BS16 1QY, UK; chisa.onyejekwe@uwe.ac.uk

**Keywords:** COVID-19, pandemic, racialised minority populations, minority ethnic, BAME, experiences, perspectives

## Abstract

Racialised minority populations were disproportionately affected by COVID-19 and saw the highest rate of COVID-19 infections and mortality. Low socioeconomic status, working as frontline workers, temporary employment, precarious immigration status and pre-existing medical conditions were factors that contributed to disadvantaged experiences. This systematic review looked at the impact of COVID-19 on racialised minority populations globally, recognising their experiences, perspectives and the effects on their physical and mental health. Eight electronic databases were searched (MEDLINE, PsycINFO, Cumulative Index to Nursing and Allied Health Literature (CINAHL), Social Sciences Citation Index (SSCI), Social Policy and Practice (SPP), Applied Social Sciences Index and Abstracts (ASSIA), MedRxiv and Research Square) for English language qualitative studies. Reference lists of relevant literature reviews and reference lists of articles were hand-searched for additional potentially relevant articles. Duplicates were removed, and articles were screened for titles and abstracts, followed by full-text screening. The Mixed Methods Appraisal Tool (MMAT) was used to assess the quality of the included studies (*n* = 70). Data were synthesised using thematic synthesis. Seven major and three minor themes were identified. The major themes related to (i) children and young people’s experiences of COVID-19; (ii) exacerbated pre-existing disparities relating to income, employment and housing security, health insurance and immigration status; (iii) lack of knowledge and information about COVID-19 and COVID-19 misinformation; (iv) racial history of medicine and treatment of racialised populations; (v) contemporary experiences of racism; (vi) impact on physical and mental health and wellbeing; (vii) concerns about safety at work. Minor themes related to (a) experiences of intercommunity mutual aid; (b) adherence to preventative guidance/COVID-19 restrictions; (c) the role of faith. Research needs to focus on developing and testing interventions that support transformation of social, cultural and economic systems towards equity of access to healthcare and healthcare knowledge. Research should be cognisant of interventions that have worked in shifting the equity dial in the past, implement these and use them to inform new approaches. Policy and practice should be mechanisms for enabling the implementation of interventions.

## 1. Introduction

Racialised minority populations (the term racialised minority population refers to groups historically treated as a minority through social processes of power and domination because of their racial identities) have been disproportionately negatively affected by COVID-19 [1,2,3]. Racialised minority populations have seen the highest rates of COVID-19 infection and mortality and have been more likely to receive ventilation interventions and suffer more serious illness [4,5,6,7]. Additional risk factors for racial minority populations include low socioeconomic status, pre-existing medical conditions and/or disability [7] and precarious immigration status [8,9,10]. Children, young people [11,12,13,14,15] and frontline workers [7,16] from racialised minority populations experienced the impact of COVID-19 in particular ways, and Black and colonised populations experienced the highest rates of hospitalisation, infection and mortality [3,5,6,17,18]. The use of the term Black, Asian and Minority Ethnic (BAME) has been criticised for suggesting homogeneity of diverse sets of different populations and can mask disparities between different communities [19,20]. We recognise the ways in which the term is exclusionary to some minority populations because it can be used as a catch-all term and is not expressive of any identity. In this review, the term racialised minority is used as an umbrella term for the many different and diverse racially minoritised populations that make up the participants from the research studies included in this review. Those different and diverse racial identities have been recognised and fully expressed through the review’s search strategy key words and that is reflected in the illustrative quotations that evidence the results. BAME appears in the review in the data extraction, reflecting how the included paper referred to research participants.

In the 2 years from the beginning of the COVID-19 pandemic, several published studies emerged about the experiences and/or perspectives of COVID-19 on racialised minority populations, but overall, the evidence was fragmented [5]. In the last 2–3 years there has been a steady growth in published research, and especially research focused on experiences and perspectives, although the current body of work lacks coalescence. This systematic review has therefore performed the important work of bringing together and integrating the existing and emergent evidence based on experiences and perspectives carried out over the last 5 years. It serves as an extensive and comprehensive archive of significance to those interested in or advocating and working towards equity of access to healthcare and healthcare knowledge.

This systematic review looked at the impact of COVID-19 on racialised minority populations globally, recognising experiences and perspectives on both physical and mental health and identifying the conditions that are needed for sustained better health and wellbeing outcomes. The review focuses on racialised minority populations and does not compare outcomes with majority populations.

Following on from the above, this systematic review had the following research questions:

1. What are the experiences and/or perspectives of COVID-19 on racialised minority populations?
2. What perceived impact do these experiences have on the physical and mental health of racialised minority populations?
3. What is perceived necessary to enable better health outcomes for racialised minority populations in relation to COVID-19?

## 2. Materials and Methods

This review followed Centre of Reviews and Dissemination (CRD) procedures [21] and was reported using the Preferred Reporting Items for Systematic Reviews and Meta-Analysis (PRISMA) guidelines [22]. The research questions and search strategy were developed using the patient, intervention, comparison, outcome and study design (PICOS) process [21]. The review protocol was registered with in the International Prospective Register of Systematic Reviews (PROSPERO) database on 16 April 2021. The registration number is CRD42021243775.

### 2.1. Inclusion and Exclusion Criteria

The inclusion criteria were as follows:

1. Qualitative or mixed methods studies (where there is a clearly defined qualitative component).
2. Studies investigating the experiences and perceptions of racialised minority populations in relation to COVID-19.
3. Peer reviewed studies.
4. Publication dates were restricted from November 2019 to May 2024.
5. Studies published in English.

The exclusion criteria were as follows:

1. Purely quantitative research.
2. Studies not about experiences or perceptions of COVID-19.
3. Not about racialised minority populations.
4. Studies where it was not possible to distinguish majority ethnic population experiences from minority ethnic population experiences.
5. Grey literature (e.g., commentaries; opinion pieces; editorials; conference abstracts; non-peer reviewed—for example, reports; book chapters; magazine articles; etc.).
6. Literature reviews.

### 2.2. Electronic Search Strategy

The following eight electronic databases were searched from the first week of November 2019 to May 2024: MEDLINE, PsycINFO, Cumulative Index to Nursing and Allied Health Literature (CINAHL), Social Sciences Citation Index (SSCI), Social Policy and Practice (SPP), Applied Social Sciences Index and Abstracts (ASSIA), MedRxiv, and Research Square. This period covers a year before COVID-19 vaccinations were widely available and approximately 18 months post the World Health Organisation (WHO) calling an end to the pandemic on an international level. Whilst this period spanned different COVID-19 variants and a period when no vaccines were available, the themes that emerged from the later research papers corresponded with those published during the earlier periods. This may be reflective of time taken to bring studies to publication. Different countries had different periods of lockdown, vaccine availability and circulating COVID-19 variants. Not all included papers had local contextualised details of timelines, so it was not possible to report on how data collection dates related to periods of vaccine development, vaccine availability and dominant COVID-19 variants.

Comprehensive pre-planned search strategies similar to that in Table 1 were designed dependent on the electronic databases listed above and their individual Medical Subject Heading (MeSH) terms. The MeSH terms used are reported in italics and key words with truncation, where appropriate. All key words and combinations were the same throughout the database searching.

### 2.3. Other Sources Searched

Reference lists of relevant literature reviews and the reference lists of articles included in this review were hand searched for additionally potentially relevant articles. All corresponding authors of the included articles were contacted to identify other potentially relevant articles missed from the electronic searches.

### 2.4. Study Screening and Selection

The screening process was conducted in three stages: 1. duplicate removal, 2. screening titles and abstracts, 3. full texts screening. The results of the electronic database searches were exported into excel where duplicate articles were removed. Handsearching of the excel file was also conducted to check for additional duplicates not removed by Excel’s duplicate removal process. Multiple publications from the same study were assessed for data duplication and, where necessary, excluded.

**Figure 1:**
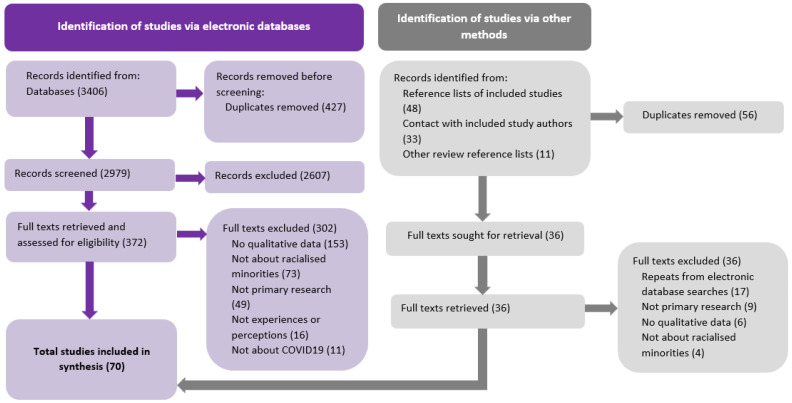
PRISMA 2020 flow diagram [22] showing the process of article identification and selection.

Title and abstract screening were completed independently by at least two review authors. Discrepancies were resolved by discussion or with the intervention of a third author. Articles that appeared to meet the inclusion criteria based on their titles and abstracts were obtained for full-text review. Where it could not be determined from the title and abstract whether an article was relevant or not these were also obtained in full-text review. As with the title and abstract screening, full-text articles were independently examined by at least two review authors. Where there was uncertainty about inclusion, consensus was reached by discussion. Reasons for exclusion were recorded and entered into a PRISMA flow diagram (Figure 1). This was also used to report the number of records identified, full-texts retrieved, full-texts included and excluded, and the reasons for full-text exclusion.

Finally, review authors checked the reference lists of the included studies and contacted the corresponding authors of the included studies for any further relevant records not identified through other sources.

### 2.5. Data Extraction and Management

Data from included articles were extracted using data extraction forms and subsequently entered into standardised tables. Data extracted included, for example, author details; year published; participant demographic details; sample sizes; and key findings related to the review research questions.

### 2.6. Quality Appraisal

The quality of included studies was assessed independently by two review authors using the Mixed Methods Appraisal Tool (MMAT) [23]. Differences in quality ratings were discussed and consensus achieved. Quality scores were not used to exclude studies but were used to identify their strengths and weaknesses.

The MMAT was chosen as it has the option of appraising multiple types of study design, including both qualitative and mixed methods studies. As mixed methods studies were eligible for inclusion in this review if their qualitative components fitted the inclusion criteria, the MMAT was an appropriate choice. The quality scores resulting from the MMAT are star (*) ratings ranging from 0 to 4, which can also be reported as a percentage value ranging from 0 to 100, were chosen for this review. Scoring was conducted by assigning a ‘yes’, ‘no’ or ‘can’t tell’ response to each of the five questions for each study type. A ‘yes’ would result in 2 points, and a ‘no’ or ‘can’t tell’ in 0 points. The scores were added up to a maximum of 10 which was then converted into a percentage score; for example, a study that scored 8 points was given a quality score of 80%.

### 2.7. Data Synthesis

Data were synthesised using thematic synthesis [24]. Data were coded line by line relative to content and meaning by one member of the team (TW). Each study’s coding consolidated or added to existing codes, allowing for synthesis across the studies. Two other members of the team (HK & RS) reviewed and provided feedback on the emergent themes. Twelve themes were coded at the initial stage and distilled to ten (seven major, three minor) after review. The initial coder then wrote up draft findings, interpreting the themes with the review questions in mind. The two reviewers offered further refining feedback on the draft findings until a final version was agreed.

## 3. Results

The electronic searches revealed a total of 3406 articles before duplicate removal. Following duplicate removal, 2979 unique titles and abstracts remained. These titles and abstracts were then screened against the review inclusion criteria by at least two of the review authors, resulting in the retrieval of 373 full-text articles.

From the above electronic database searches, two literature reviews relevant to the topic of racialised minorities and impact of COVID-19 [25,26] were retrieved and their reference lists screened for studies possibly fitting the inclusion criteria. This revealed a further 11 potentially relevant articles. After screening titles and abstracts, two full texts were retrieved but were excluded as they did not investigate experiences or perceptions of COVID-19. Reference list searching of articles already included in the synthesis led to the identification and screening of a further 48 titles and abstracts. Of these 48, 23 full texts were retrieved, but none fitted the review inclusion criteria. This was largely due to the studies not specifically investigating experiences or perceptions.

In total, 70 studies fitted the inclusion criteria and were included in the synthesis. Full details of the process of including and excluding articles, with reasons, is presented in Figure 1.

### 3.1. Included Study Details

The majority of the included studies (83%) were from the United States of America (USA) at 33 (47%), the United Kingdom (UK) at 18 (26%), and Canada at seven (10%). The remaining 12 studies (17%) were primarily from Europe, but there were inclusions from Chile, Hong Kong, New Zealand and the United Arab Emirates (UAE), at one study each. Most studies were published in 2021 (39%), 2022 (21%) or 2023 (27%), highlighting a rapid response from academics to explore the impact of COVID-19 on racialised minorities. At the time of the final electronic database searches for this review in May 2024, six studies (9%) fitting the inclusion criteria had been published, showing research activity is ongoing and evolving on this topic. The majority of included studies did not report where the research was conducted with urban or rural populations. However, in the studies where the setting was stated, most were in urban areas (19%), with smaller numbers in rural areas (9%), or both urban and rural locations (10%). Further information on included study details is available in Supplementary Table S1.

### 3.2. Participant Demographic Characteristics

The 70 included studies contained combined participant numbers of 3326. Participant gender was not reported in seven studies. Of the remaining 63 studies, there were 2054 females, 954 males, 29 transgender, nine non-binary, and three described as ‘other’. The age of participants was sporadically reported, with 40 included studies not reporting the mean age, 25 not reporting the age ranges, and 62 not reporting the median age. However, in the studies where ages were reported, there was an overall mean age of 38.8 years, an overall age range of between nine and 93 years, and an overall median age of 37 years.

The religion of participants was not reported in most of the included studies. However, in the eight (11%) where it was reported, there were 119 Muslim, 112 Christian, 14 Catholic, 3 Hindu, 5 described as ‘other’ and 17 participants with no religion. Ethnicity was described in all 70 included studies; however, in four of them, there was no breakdown of numbers of participants by ethnic group. For the remaining 66 studies, the combined ethnicity of 3158 participants was Black/African—1031, White/European—574, Asian—536, Latin—650, Arabic—120, Roma/Gypsy—107, Indigenous peoples—56, mixed—28, other—56. For a detailed breakdown of participant characteristics, please see Table S1.

### 3.3. Methods and Quality Scores of Reviewed Studies

Sixty (86%) of the studies included were qualitative [8,9,12,13,27,28,29,30,31,32,33,34,35,36,37,38,39,40,41,42,43,44,45,46,47,48,49,50,51,52,53,54,55,56,57,58,59,60,61,62,63,64,65,66,67,68,69,70,71,72,73,74,75,76,77,78,79,80,81,82], with the remaining 10 (14%) being mixed or multiple methods [83,84,85,86,87,88,89,90,91,92]. All but two (97%) studies were cross-sectional, with the remaining being longitudinal [89,92]. A variety of sampling procedures were used, with 28 purposive [8,9,13,27,30,31,33,34,35,43,45,46,48,49,50,55,63,67,72,74,78,79,81,83,85,90,91,92], 13 convenience [29,32,51,54,58,66,75,76,77,80,84,87,88], 5 snowball [28,38,41,64,65], 2 random [86,89], and 22 using combinations of the three [12,36,37,39,40,42,44,47,52,53,56,57,59,60,61,62,68,69,70,71,73,82]. As would be expected with qualitative research, most data collection was conducted through participant interviews, with 50 studies using this method [8,12,13,28,31,32,33,34,35,36,38,39,40,42,43,45,46,47,48,51,52,53,54,55,56,57,58,59,60,61,62,63,64,65,66,67,71,72,73,74,75,77,78,80,81,82,83,86,88,90], 10 studies using focus groups [9,27,29,30,37,41,50,69,87,91], 7 used both methods [32,45,49,68,70,79,92], and 4 using surveys [44,76,85,89]. Data analysis methods were more varied; however, most (38) used thematic analysis [9,12,13,27,28,29,30,31,33,35,38,42,43,44,45,50,51,53,57,59,61,64,65,66,67,68,69,70,74,76,77,79,80,81,82,83,87,89], with smaller numbers (4) using coding [47,75,84,91], grounded theory procedures (3) [8,32,78], or constant comparative analysis (2) [34,71]. Full details on included study methods are available in Table S2.

Study quality scores ranged from 4 (40%) to 10 (100%) out of a maximum of 10 (100%) using the Mixed Methods Appraisal Tool (MMAT) [23]. Overall, study quality was high, with 50 of the 70 included studies scoring the maximum 100% [9,12,13,27,28,29,30,31,33,34,35,36,38,39,41,42,43,45,46,47,48,49,50,51,53,54,55,56,58,59,60,62,64,67,68,69,70,71,72,73,74,76,78,79,80,81,82,86,87,90], and a further 11 scoring 80% [8,32,61,65,66,75,77,83,85,88,91]. Details of study quality scores are available in Table S2.

### 3.4. Thematic Synthesis of the Included Studies

Seven major and three minor themes were identified from the data synthesis of the included studies. Major themes had substantially more data relating to them and appeared across more of the included papers, whereas minor themes were relevant, but less data emerged about them, and they appeared less frequently across the included papers. The major themes related to (i) children and young people’s experiences of COVID-19; (ii) exacerbated pre-existing disparities relating to income, employment and housing security, health insurance and immigration status; (iii) lack of knowledge and information about COVID-19 and COVID-19 misinformation; (iv) racial history of medicine and treatment of racialised populations; (v) contemporary experiences of racism; (vi) impact on physical and mental health and wellbeing; (vii) concerns about safety at work. Minor themes related to (a) experiences of intercommunity mutual aid; (b) adherence to preventative guidance/COVID-19 restrictions; (c) the role of faith. Data were mapped according to ethnicity and race to see if some themes related to particular groups, but this was not the case. There was a broad representation of different racialised minority populations across all the themes (see Table S1 for ethnicity and/or nationality of participants from included studies). The results are underpinned by indicative quotations from across the representative populations for each theme in Table 2.

### 3.5. Children and Young People’s Experiences of COVID-19

The impact of COVID-19 on children due to school closures was mostly negative, with some positives. Limited access to online schooling negatively affected developmental and academic skills and lack of usual social networks for children adversely affecting mental health, including for children with special educational needs and disability. There was also reduced physical activity due to school closures and living in small properties. With free school meals inaccessible, a lack of money and reliance on food banks, eating habits changed and the quality of food that children ate was reduced. Parents and carers struggled to balance work and home schooling and keep children occupied when they had limited technological infrastructure, hardware and skills. Technology sharing was a cause of conflict in families. Gambling and substance dependency worsened, and school dropouts increased. A lack of education was seen as a barrier to a better future. Parents and carers’ loss of employment and financial hardships meant children lacked basic essentials like food and clothing. For adolescents, there was sleep disruption, angry outbursts, lethargy, sadness and confusion and fear of the virus, and a general sense of helplessness about the world during lockdown as they felt cooped up, restless and bored. There were also experiences of unfair treatment and discrimination in schools and on the job market for young people. Some positives for children and young people included more time spent with family and on themselves, which increased parent/carer child bonding. Those with gardens and larger homes or access to outdoor spaces enjoyed more physical activity, and there was development of technological skills because of the transition to online schooling. Where therapy services went online, this was helpful for children and young people, although no detail was provided as to how this was the case. In terms of what is needed to enable better health outcomes in relation to COVID-19, data from Albanian adolescents showed that a societal shift in attitudes towards young people was needed for them to benefit from a better future.

### 3.6. Exacerbated Pre-Existing Disparities

Pre-existing health and social disparities for racialised minority populations, relating to income, employment and housing security, health insurance and immigration status, and other experiences of oppression and unjust systems, were exacerbated during the pandemic, meaning a greater susceptibility to infection and worse outcomes for those populations. Financial uncertainty came from loss of employment and caused hardship, and consequently housing insecurity and food rationing. Precarious immigration status meant ineligibility for government support payments in the USA and even if people were able to access government financial support, their future rights to a green card in the country were negatively affected. Lack of health insurance, in the USA, led to concerns about being excluded from vaccine programmes. Lack of funds from unemployment or low-wage employment prevented people travelling to vaccine centres and meant an inability to afford personal protective equipment. Lack of technologies made accessing healthcare professionals/services and medications challenging. Working in low-wage economies often also meant working on the frontline and in close proximity, which meant adhering to COVID-19 restrictions was challenging. These factors, along with large intergenerational households often living in overcrowded small, confined housing, also meant adhering to COVID-19 restrictions was further challenged, resulting in higher rates of infection. The perspective for Latino populations was that individuals were responsible for employment struggles and housing precarity. There was a sense that attributes such as self-reliance, self-protection and forbearance were required to tackle social and health disparities. Having a higher social economic status, such as some Canadian Asian populations, was a protective factor. They benefitted from more assistance from family and greater financial support, but this was related to individual circumstances, such as profession or level of income, rather than culture or ethnicity. In terms of what is needed to enable better health outcomes in relation to COVID-19, there are learning points to be gained. The pandemic shone a light on pre-existing disparities and global inequities. It illustrated that intersectional approaches that draw on the work of past movements are important for improving inequities. Also reported was frustration with the broader positive notion that all-inclusive vaccination was needed as a mechanism for facilitating society getting back to ‘normal’. This was not needed and instead was viewed as problematic for racialised minority populations, as returning to ‘normal’ does not solve pre-existing disparities, it merely sustains them.

### 3.7. Lack of Knowledge and Information About COVID-19 and COVID-19 Misinformation

A lack of information and knowledge about the COVID-19, thin networks or knowledge that the vaccine was free led to apathy about the virus and being vaccinated. The information gap was plugged by word of mouth, social media, religious texts and family and friends, so combinations of information sources were used, although social media was a large information provider. Younger generations and community and religious networks especially filled knowledge gaps, but sometimes this led to perceptions of low risk. Language barriers, illiteracy, including digital illiteracy, resulted in a lack of information getting through, and that lack of information and knowledge led to higher infection rates. These factors were particularly relevant for older people. There were experiences of confusion about information and conflicting information from all sides and that made it difficult to identify what were trusted forms of information. The US president was especially considered an untrustworthy source of information. At times, some doubted the existence of the virus or that it was a risk for them. There was frustration about information coming from health officials about best practices, COVID-19 and COVID-19 vaccine misinformation that led to confusion, misunderstanding and distrust. In terms of what is needed to enable better health outcomes in relation to COVID-19, overcoming vaccine hesitancy requires populations to do further trusted reading, information and actions from friends and family, especially those who happen to be healthcare professionals. Trusted groups need to be in place to provide and communicate information. Community initiatives also need to be set up to counter the lack of information and provide practice support.

### 3.8. Racial History of Medicine and Treatment of Racialised Populations

There was mistrust of medical professionals and the state, experiences of government failure, systemic racism and previous negative healthcare treatment from across most groups, all of which prevented people from taking up the COVID-19 vaccine. Past and present healthcare experiences included medical apartheid, poor treatment, discrimination and condescension from healthcare staff and marginalisation and neglect from emergency services, which meant avoidance of hospitals and scepticism that the COVID-19 vaccine was being given to racialised minority populations first as a form of experimentation. Mistrust of authoritarian government systems from countries of origin was a barrier to uptake of preventative measures and there were fears of institutions as agents of control. Further, the COVID-19 vaccine testing systems and pandemic restrictions were seen as mechanisms to track and control certain populations or that it could be used for genocide. People were aware of past poor and unethical medical research practices involving racialised minority communities and therefore mistrusted the vaccine because it had been developed in a rush. Based on knowledge and personal experience, there was feeling that the vaccine had not been developed with minority populations in mind and worry that the effects for them would be different. There was a sense of frustration that racialised minority communities had been overlooked and that there had been systemic delays in them being able to access vaccines. Alongside this, there was a lack of reaching out to racialised minority communities to understand the reasons for vaccine hesitancy. Some populations in the USA (African Americans and Latinx) did report trust in healthcare workers and local government, but not in federal/central government, and some said they would access care despite potential barriers like precarious immigration status or lack of health insurance. In terms of what is needed to enable better health outcomes in relation to COVID-19, strategies need to be aimed at reaching overlooked communities and the most vulnerable within those communities in particular. Further, racialised minority population healthcare workers advocating and role modelling by having the vaccine themselves are important for building trust and bridging cultures.

### 3.9. Contemporary Experiences of Racism

Racism was experienced across populations. During the time of the Black Lives Matter (BLM) protests, racialised minority populations felt their physical health was also at risk. They felt threatened by police violence and White supremacists. Generally, during the pandemic there were intensified fears, anxieties and a sense of worthlessness because of experiences of racism. Overt discrimination, racial motived attacks and covert microaggressions, vicariously, in-person and online were reported, leading to feelings of hopelessness, depression and the avoidance of public space as a strategy to circumvent incidents. Public spaces were viewed as unsafe spaces because of racially motivated attacks, and this made people feel anxious, scared, depressed, angry and outraged. There were reports from Gypsy, Roma and Traveller communities of post not being delivered when COVID-19 cases on site were known about, leading to hospital appointments being missed. Metal barriers were also put up across site entrances which prevented ambulances from accessing sites in emergencies. The psychological and emotional impact of discrimination caused depression, hopelessness, inability to form meaningful relationships and persistent stress. Experiences of being bullied and racially discriminated against at work were reported. Managers put racialised minority population workers at higher risk from COVID-19 whilst white workers were shielded to protect their older relatives on assumption that racialised minority population workers did not have family living in the country. Personal protective equipment was not ethnically targeted or appropriate, and technologies for homeworking were given more often to white colleagues. Workloads for racialised minority population staff were increased whilst white workers were signed off sick or took leave. There was no workplace recognition of the murder of George Floyd and the BLM protests. Staff felt that work-based risk assessments were treated as a tick box exercise and not a protective mechanism for those at higher risk from COVID-19. There was a longing for normalcy and hope reported. In terms of what is needed to enable better health outcomes in relation to COVID-19, more speedy policy changes are needed. Rapid policy changes were made during the pandemic for the provision of safe housing, and this led to quick health improvements and dispelled the myth that policy change must be incremental and not radical and fast. Protest and direct action do have an impact. There were reports of behaviour change in white people who engaged in dialogue about racism because they noticed it happening, especially in light of the BLM protests. Government support and campaigns for racialised minority population healthcare workers are important for people to feel valued and recognised. Crisis planning ahead of emergencies for the needs of vulnerable populations is more beneficial than planning in a stressful time of heightened risk. There was a spreading of misinformation, blame culture and incitement to hate, especially towards Asian people, by political leaders and mainstream media, which was upsetting. Discrimination and stigma came through the misrepresentation of communities in the media and messaging that blamed racialised minority populations for the spread of the virus, which increased stigmatisation and alienation. Discrimination was experienced through a lack of support for racialised minority communities in the face of COVID-19 and its effects. Stereotyping around the virus origin and the link between racialised minority communities prevented people getting tested and diagnosed and resulted in internalised racism. There was discriminatory monitoring of racialised minority populations around restrictions.

### 3.10. Concerns About Safety at Work

Doctors in particular reported positive work environments and a sense of feeling recognised, rewarded and visible for their service during lockdowns, but for others there were concerns about being at/returning to work because of exposure to the virus from frontline jobs. Working on the frontline did not allow for homeworking and was a social economic barrier to adhering to restrictions, which in turn meant a higher risk of infection. Lower social economic status meant increased chances of working on the frontline and in close proximity to others, leading to increased infection rates. There were incidents of legally mandated work breaks being broken, meaning care staff were constantly pulled into performing more of their frontline work. Informal carers tended to be in frontline paid employment, meaning the risks of infection were increased for them and the vulnerable family members they cared for. On top, the supply of personal protective equipment for frontline workers was minimal, inconsistent and inadequate, and there was peer pressure to sometimes not use it, putting care workers at more risk. Some ended up buying their own supplies or isolated themselves from family. Workers felt pressure to continue frontline work and put themselves at risk as they felt they were sometimes unsupported by managers to make other decisions, as well as for financial and personal reasons. Permanent workers had more options to take paid leave and sick leave, although some recounted that managers made decisions that meant Black staff were more often the ones at work, covering for those shielding at home. Mixed experiences of working at home were reported. Money was saved on travel expenses, but there were some personal costs to be borne, such as using their own phone and utilities. Also, there was a sense of feeling monitored by managers and harassed to return to the workplace after periods of COVID-19 sickness. There was a sense that COVID-19 guidance was aimed at white collar privileged workers and not reflective of the work racialised minority communities were engaged in.

### 3.11. Impact on Physical and Mental Health and Wellbeing

Mental health and psychological wellbeing were negatively affected, and existing mental health challenges intensified. There were experiences of emotional distress such as nervousness, anxiety, stress, tiredness and sadness and hopelessness from infection exposure, confinement, online schooling, juggling work and relationships and lack of support. Anxiety increased, along with depression, isolation and altered sleep patterns and lack of sleep. A lack of internet access and living in such close proximity to family/community meant tensions rose, and this was especially so when families/community showed a lack of understanding about mental illness. Mental health was also affected by a lack of culturally competent services and there were experiences of loss and separation from friends/family/communities/social groups. There was also a sense of being overwhelmed by sudden unexpected deaths and an emotional toll from grief.

Anxiety happened amid the sudden and ongoing changes, and there was a sense of disconnection from communities because of the restrictions. Worry over financial hardship and providing for families meant work was prioritised over risk of catching the virus. There was also fear that vulnerable individuals in racialised minority communities, such as Special Educational Needs and Disabilities (SEND) children, older people and people with pre-existing conditions, such as sickle-cell anaemia, HIV or dementia, would and did experience worse health outcomes. They were also negatively impacted by informal carers not being allowed to be present in clinical settings. Informal carers worried about the complexities of becoming unwell themselves from the virus or the vaccine and not being able to care. They took extra precautions such as sanitising food or self-imposed stricter lockdowns to ensure risks were lowered. Informal care work increased and led to feelings of isolation as shared and/or paid carers/allied services were cancelled/closed. This meant there was a lack of time for self-care for informal carers. Frontline workers feared infecting family members at home and so took additional extreme risk reducing hygiene measures. Transgender men and women were concerned about accessing gender-affirming hormones and procedures because of delays in treatment and in the increase in hormone costs. Those working in low-wage economies felt their physical health was negatively impacted, causing persistent pain due to lack of sick pay and health insurance. Workers worked through and did not take time off. Some reported feeling bullied or coerced into having the COVID-19 vaccine. Loneliness and feelings of isolation were reported when working from home.

There was worry about what was happening where people were, but also in countries of origin where friends and family were. Concern, frustration and sadness also existed around how governments in countries of origin were responding to the pandemic, and whether people from ancestral countries were taking the virus seriously and if healthcare systems there could cope. Contact with family did alleviate anxieties for some. There was some concern about the impact of the pandemic on the global economy and on underdeveloped countries.

On the positive side, staying home meant feeling safe from COVID-19 and there were benefits such as more time to spend and bond with family and friends and enjoy leisure activities, with less stress due to not having to carry out the school run/morning rush.

### 3.12. Intercommunity Mutual Aid

Local community-based organising filled the support gaps left by governments. This included setting up food banks and befriending projects. Information was shared across communities and communities acted as translators and sorters of information. Online gatherings with friends and family were important forms of social support, for example, an LTBTQ bubble created to maintain connections and support for African Americans and Latinx LGBTQ folks.

### 3.13. Adherence to Preventative Guidance/COVID-19 Restrictions

Cultural norms around gatherings superseded social distancing guidance and understanding of infection being more likely. This made maintaining social distance challenging, although some populations took action to reduce risks, such as use of sanitisers and social distancing. There was an avoidance of human and health services by some so as to not be exposed to the virus. Government officials flouting restrictions led to questioning of the necessity for restrictions and guidance, although some populations felt local government and community leaders encouraged adherence and reinforced the need to follow guidance.

### 3.14. The Role of Faith

Whilst not all faiths were accounted for in the literature, there was significant information about those from Christian and Muslim faiths. There was a sense of loss from not being able to access church and faith groups/community, and for clergy frustration from not being able to support parishioners and the sick. Important religious and cultural events were cancelled, and funerals curtailed. Where well-attended funerals were a mark of respect, it was upsetting they could not happen. Faith was key to community resilience, although also led to belief in divine causation. It was also key to overcoming fear and anxiety. There was a sense that illness was a sign of weakness, or a fatalistic sense that having the virus, or not having it, was a sign from/the will of God, particularly for those from Christian and Muslim faiths. Home remedies for COVID-19 were important and linked to cultural and religious practices. In terms of what is needed to enable better health outcomes in relation to COVID-19, church and faith communities are important key links for reaching overlooked/underserved people.

## 4. Discussion

The purpose of this review was to look at the impact of COVID-19 on racialised minority populations, recognising their experiences and perspectives affecting both physical and mental health. COVID-19 has revealed and exacerbated the pre-existing racial and socioeconomic disparities that have disproportionately impacted the health outcomes of racialised minority populations driven by a complex interplay of socioeconomic determinants of health and deep-rooted structural inequalities [93,94].

Factors such as pre-existing health conditions, multi-generational living arrangements, confined or overcrowded housing, occupation, immigration status, trust in institutions and sociocultural beliefs towards illness and vaccination affected racialised minority populations’ experiences and perspectives of COVID-19. Language barriers, digital illiteracy and a longstanding mistrust resulted in limited access to accurate and consistent information that hindered effective communication, fuelling misinformation about COVID-19, including vaccine hesitancy [95,96].

Children and young people experienced school closures, impacting their physical activity, academic performance and social skills, with those from disadvantaged backgrounds hit hardest, having free school meals stopped and limited access to technologies for online schooling, putting them at additional risk of food insecurity and disadvantaged futures [97,98,99]. Parents and carers from racialised minority populations struggled to balance work and home schooling as they were disproportionately represented in frontline roles within health and social care and other high-risk public-facing occupations, including roles in retail and transport, placing them at increased risk of COVID-19 exposure, further compounding their vulnerability and exacerbating the inequalities they faced [100,101,102,103]. For some children and young people, the increased time spent together with families strengthened connections, fostering better understanding and stronger emotional bonding. However, socioeconomic determinants such as loss of employment caused financial hardships, increasing housing insecurity, food rationing and conflict within families.

Deep-rooted structural inequalities contributed to the racialised minority populations being racially discriminated against at work, including increased workload to cover sickness of white staff, lack of opportunity to work from home and pressure from managers to continue working in frontline services to protect their employment and ensure economic sustainability to avoid financial hardships [104,105]. Lack of effective guidance protecting racialised minority populations, spreading of misinformation, blame culture and incitement to hate, backed by some key political leaders and mainstream media, intensified fears and anxieties, making them more vulnerable and marginalised.

The physical health of racialised minority populations, especially those working in frontline services, led to increased exposure to COVID-19, leading to consistent and worse symptoms linked to comorbidities and higher death rates [106,107]. The impact of COVID-19 on mental health was multifaceted and led to increased anxiety and emotional distress due to low socioeconomic circumstances, raised risks of infection, fears of passing the virus on to families for frontline workers, loss of loved ones, social isolation, experiences of racism and discrimination, and barriers to accessing culturally sensitive mental health services [108,109,110]. In addition, racialised minority populations experienced anxiety and concern for families and loved ones residing in their countries of origin, where healthcare services were often under-resourced and limited. Travel restrictions and the spread of misinformation further added to the frustration, helplessness and emotional distress, exacerbating their mental health problems.

Although majority populations living in lower socioeconomic backgrounds experienced similar disadvantages during the COVID-19 pandemic, the intersection of economic hardship with other cultural and structural factors, such as pre-existing racial disparities, inadequate living conditions, migration status, and systemic inequalities, further exacerbated the marginalisation of racialised minority populations. These intersecting forms of disadvantage compounded their vulnerability to both the health and socioeconomic impacts of the pandemic, amplifying existing inequities and limiting access to essential resources and support.

This review has highlighted the need for the standards of healthcare to be raised for racialised minority populations, in accordance with the wider determinants of health for sustained equity and support, so that when the unexpected happens, racialised minority populations do not face amplified challenges.

### Limitations

We looked at racialised minority populations across the globe but did not consider where those populations may be in a majority but subject to oppressive and/or marginalising systems, such as in South Africa. The overwhelming majority of the articles reviewed were from the United States of America, the United Kingdom and Canada. Although geographically distanced, these countries share a similar baseline of social care and healthcare opportunities, although the USA reflects some differences due to the insurance healthcare model.

It is important to recognise that race is socially constructed and experienced differently in different countries, which may limit a global comparison of racialised minority population experiences, perceptions and outcomes. It was not our intention to present a comparison and, in this sense, when we themed the data, we cross-referenced the themes with the included papers to enable identification of country, nationality/ethnicity and local specific context commonalities. We found that the themes were fairly universal, without nationality/ethnicity or local country-specific socio-political and economic differences. This may be because most of the papers included came from Western countries.

Due to the nature of the review topic, copious amounts of studies were ongoing whilst it was being carried out. This meant new literature was being published all the time and at speed. The research team had to be responsive in re-running the searches a number of times. It meant that the review was much larger than originally anticipated.

## 5. Conclusions

Whilst the results showed some variations in the experiences and degrees of impact among racialised minority populations, the review revealed substantial commonalities in how these groups experienced and perceived the effects of COVID-19. Although not addressed in the data, country-specific socio-political and economic contexts might be a contributor to experiences, perceptions and outcomes.

To protect racialised minority populations in similar future public health emergencies, participants reported the following: societal shifts in attitudes; strategies that draw on past movements for inspiration; intersectional approaches; communication through trusted routes (including through faith communities) and reaching out to overlooked and underserved communities.

To enable this, sustainable culturally sensitive, anti-racist and anti-oppressive health policies and education programmes that centre the marginalised and oppressed, along with significant financial investment in health services that are available free for all are needed. Research can support this effort by focusing on developing and testing interventions that will support transformation of social, cultural and economic systems towards equity of access to healthcare and healthcare knowledge. Research should also be cognisant of interventions that have worked in shifting the equity dial for past generations and look to implement these and use them to inform new approaches. Policy and practice should be mechanisms for enabling the implementation of interventions.

## Figures and Tables

**Table 1:** Example search strategy used in MEDLINE.

Concept	Search Terms	
**Ethnicity**	*Ethnic Groups*; *Minority Groups*; *African Continental Ancestry Group*; *Asian Continental Ancestry Group*; *Roma*; minority ethnic; Black and minority ethnic; Black, Asian and minority ethnic; ethnic minoriti$; people of colo?r; Black, Indigenous and people of colo?r; Black African$; African Caribbean; African American$; Pakistani$; Indian$; Bangladeshi$; South Asian$; East Asian$; South East Asian$; filipin$; Arab$; mixed race$; race$; raciali?ed minorit$; duel heritage; gyps$; BME; BAME; POC; BIPOC; Irish Travel?er$; GRT; Native American$; Latin$; Hispanic; First Nation$; Global Majority	OR
AND		
**COVID-19**	*Coronavirus*; *Coronavirus Infections*; *COVID-19*; COVID; COVID19; COVID-19; 2019-nCoV; novel coronavirus; SARS-CoV-2; severe acute respiratory syndrome coronavirus 2; coronavirus disease 2019; coronavirus pandemic$; pandemic$	OR
AND		
**Experiences and perceptions**	Experience$; perception$; opinion$; attitudes$; perspective$; view$; belief$	OR

Note: $ denotes truncation; ? denotes wildcard (e.g., also searches Americanised spellings); medical subject headings (MeSH) terms are in italics.

**Table 2:** Indicative quotations. Supplementary File Table S3 shows a broader range of indicative quotations.

Theme	Indicative Quotations
Children and young people	“The situation became very difficult during the pandemic because my father had no job, my mother was sick, and we had no money to buy medicine or bread. We were very scared as we did not know how long this situation would last” (Egyptian and Roma Albanian) [87] “Not being able to play in my basketball team, this makes me really angry, sad, and depressed” (African American; Latinx; Asian American; American Indian; Other/Mixed) [89]
Exacerbated pre-existing disparities	“They don’t need to put the word documents [in COVID vaccine adverts] because. what if I don’t have it, I’m undocumented. And you said okay come on have your vaccine, we’re not going to check you. I won’t go because I don’t know to what extent is true. It might be a ploy to get people to come” (African; Eastern Mediterranean; Sri Lankan; Venezuelan) [36] “Indigenous peoples statistically have high rates of respiratory and heart disease, due to a variety of longstanding determinants of health stemming from continued colonization. These conditions make a person especially vulnerable to COVID-19” (Canadian Indigenous) [66]
Lack of knowledge and information about COVID-19 and COVID-19 misinformation	“The elderly people around me have not gotten vaccinated because the sites of vaccination are too far away”. “We don’t read English, and we don’t know how to use the Internet well, so it’s difficult” (Latinx; Black/African Americans; Chinese Americans) [32] “For me, I would like to take the vaccine if that will make everything better. But the fake news is scaring me, so I don’t know. That is a problem. I don’t know if it’s real, I don’t know if it’s fake. When you take it, it will change the DNA… it will stop the person not having kids in future. A lot of stories are flying” (African; Eastern Mediterranean; European; Sri Lankan; Venezuelan) [36]
Intercommunity mutual aid	“I truly believe that Roma women can do whatever we want, because during the lockdown, many Roma women created ourselves a food bank. The work we have done is huge, and many other Roma and non-Roma organizations have joined us in our effort to guarantee basic needs for Roma families. We helped almost 350 Roma families, so definitely we can achieve whatever we propose to do” (Roma) [27] “We have, as a Somali community, determined together to assemble the folks and communicate to our community the best and most reliable information” (Somali) [38]
Racial history of medicine and treatment of racialised populations	“To be very honest—and it’s getting back to a whole lot of things that have happened to our people back in the day. They don’t trust doctors. They don’t trust people…I haven’t taken the test. I don’t know if I’ll take the test” (African American) [30] “My neighbors say ‘No’ [to getting the vaccine], because they [the government] are going to put a chip in them, or because they might put another virus [in them]… that’s what people from my community think.” (Latinx and Purépecha) [41]
Contemporary experiences of racism	“Trump saying that it’s the China virus—it’s obviously we cover so much US politics in Canada that I think it’s influenced the crazy people in our country as well. Or it’s resonated with them, I guess.”……It’s just that the mental impacts of it can be sometimes really overwhelming.” “All of the Asian hate crimes are scary, in my opinion. I am less willing to do things alone and go out into neighborhoods by myself” (East Asian; South Asian; Southeast Asian; West Asian) [57] “(Anonymised person) I know your parents are in [Country name] in Africa, so you won’t have grounds for shielding.” “You can tell the unease arising from BLM for some of our white colleagues……”Risk assessments introduced for Black staff were experienced as a tick box exercise and not as a protective mechanism for those at higher risk of COVID-19. “my risk assessment was done virtually. My manager asked if I felt well and healthy, whether I had (you know) any existing health conditions, I said no and that was it.” (Black African) [77]
Adherence to preventative guidance/COVID-19 restrictions	“In the Eastern culture, social contact is a very important aspect of our daily routine. I can’t spend a day without meeting with my social network. In my view, I would have preferred to get the coronavirus infection rather than being isolated” (Somalia; Iraq; Pakistan; Afghanistan, Poland; Sri Lanka; Turkey; Bosnia/Serbia; Eritrea; Syria) [42] “I have been extremely social distancing. I, in fact, haven’t left my house; some of my friends do the grocery shopping for me and leave my groceries by the door. I bring them inside, sanitize them, and throw the bags out” (African American; Latinx) [71]
Impact on physical and mental health and wellbeing Multilocal concerns—moved to mental wellbeing	“Everything is available, but my mental health is in a very poor state. The last time I experienced similar anxiety was in 1991 in Iraq during the war.” “I can’t say where I can get help. If I got a service in my own language, then I might know who to contact. […] News [readers] speak quickly. Difficult to understand them. Finnish is the most difficult language” (Arabic; Iraqi; Syrian; Palestinian; Somali) [38] “But now what they’ve done, there’s no way could you get that lock off. They’ve fitted a box round it, so you can’t get into it. […] you’d have a heart attack and you’d be dead.” (GRT) [55] Multilocal concerns “I’m worried about my homeland. I am worried about both [countries]. We live here, and my family lives in [my] homeland” (Arabic; Iraqi; Syrian; Palestinian; Somali) [38]
Concerns about safety at work	“I think I got COVID-19 because I was using public transportation to get to work … when I would get on the bus, there were lots of people without face masks or gloves and it didn’t look like they had their own hand sanitizer either.” (Latinx) [8]
The role of faith	“I do my best, but God protects” (Arabic; Iraqi; Syrian; Palestinian; Somali) [38] “Coronavirus is punishment from God for disobedience” (African; Indian; Caribbean; Pakistani; Bangladeshi) [78]

## Data Availability

The raw data supporting the conclusions of this article will be made available by the authors on request.

## Supplementary Materials

The following supporting information can be downloaded at: https://www.mdpi.com/article/10.3390/ijerph22121767/s1, Table S1: Included study aims and participant demographic characteristics; Table S2: Included study methods and quality scores; Table S3: Indicative quotations.
