# Supplementary material for: The Impact of COVID-19 on Racialised Minority Populations: A Systematic Review of Experiences and Perspectives
Source: Int J Environ Res Public Health. 2025 Nov 21;22(12):1767. doi: 10.3390/ijerph22121767 (PMC12732843; doi:10.3390/ijerph22121767)
Supplement: Supplementary file 1 [file ijerph-22-01767-s001.zip › ijerph-3809498 - table S1.pdf]

Table S1. Included study aims and participant demographic characteristics (supplementary file).

Papers [Country]	Aims	Participant numbers	Participant type	Ethnicity and/or nationality	Religion	Age in years: Mean [Median] (Range)	Gender (%)	Urban/ rural
[8] [United States of America]	To describe the experiences of Latinx individuals who were hospitalized with and survived COVID-19.	60	General public (73% classed as essential workers)	Latinx	NR	48 NR NR	24 females (40%) 36 males (60%)	NR
[9] [United States of America]	To explore community perspectives on readiness for COVID-19 vaccination and to identify culturally tailored vaccine outreach strategies.	15	General public	Latinx	NR	41 NR (29-65+)	10 females (66%) 5 males (34%)	NR
[12] [United States of America]	To explore African American parents' experiences with using technology to engage their children during COVID-19 and the impact on family health.	11	African American families with at least one school-aged child	African American	NR	NR [43.5] NR	9 females (82%) 2 males (18%)	Rural
[13] [United States of America]	To explore the perceptions of female-headed African American families on "stressors," "stress reactions," and "opportunities" amid the COVID-19 pandemic lockdown mandates.	9	African American families	African American	NR	NR [40] NR	All females	Rural
[27] [Spain]	To gain an understanding of the key challenges the pandemic raised and its impact on Roma communities.	25	Roma women students	Roma	NR	NR NR (9-67)	All females	NR
[28] [Denmark]	To investigate COVID-19 vaccination perceptions in one of Denmark's largest ethnic minorities, the Arabic-speaking community.	16	Arabic speakers from Arabic speaking counties	Arabic: Iraq (5); Syria (3); Sudan (2); Palestine (2); Egypt (1); Kuwait (1); Lebanon (1); Algeria (1)	NR	42 [37] (22-76)	8 females (50%) 8 males (50%)	Urban
[29]	To gain a qualitative understanding of vaccine beliefs and attitudes toward	17	Somali population in the American state of Wisconsin	Somali	NR	NR NR (20-69)	9 females (53%) 8 males (47%)	Rural

Table S1. Included study aims and participant demographic characteristics (supplementary file).

Papers [Country]	Aims	Participant numbers	Participant type	Ethnicity and/or nationality	Religion	Age in years: Mean [Median] (Range)	Gender (%)	Urban/ rural
[United States of America]	COVID-19 among Somali residents living in Northern Wisconsin.							
[30] [United States of America]	To examine perceptions of COVID-19 related to prevention, coping, and testing of African American residents in under-resourced communities in Alabama.	36	Community residents and stakeholders	African American	NR	54.3 NR (29-77)	24 females (67%) 12 males (33%)	Urban and rural
[83] [United Kingdom]	To identify and gain insight into factors influencing COVID-19 vaccination amongst health and social care workers in the UK.	20 interviewed of 1917 surveyed	Health and social care workers	White British (8); Asian/Asian British (5); Black/Black British (3); White Other (2); Mixed (2)	Christian (9); No religion/ atheist (7); Hindu (3); Muslim (1)	NR NR (25-64)	16 females (80%) 4 males (20%)	NR
[84] [United States of America]	To understand how self-identified Black pregnant women in the United States were affected in the early days of the COVID-19 pandemic.	87	Pregnant African American self-identified women	African American	NR	32.4 NR NR	All females	Urban and rural
[31] (2023) [Chile]	To explore the experience and perception of international migrants in Chile regarding access to health services during the pandemic.	40	South and Central American migrants (30); Health care workers (10)	Migrant participants: Venezuela (7); Peru (5); Colombia (5); Haiti (3); Bolivia (2); Ecuador (2); Argentina (2); Brazil (2); Cuba (1); Uruguay (1)	NR	NR NR NR	16 females (53%) 14 males (47%)	NR
[32] [United States of America]	To explore knowledge and beliefs about COVID-19 vaccination among Black /African American, Latinx, and Chinese American residents of the San Francisco Bay Area and assess their	109	Marginalised populations	Latinx (40); Black/African Americans (35); Chinese Americans (34)	NR	NR NR (18-65+)	76 females (70%) 33 males (30%)	NR

Table S1. Included study aims and participant demographic characteristics (supplementary file).

Papers [Country]	Aims	Participant numbers	Participant type	Ethnicity and/or nationality	Religion	Age in years: Mean [Median] (Range)	Gender (%)	Urban/ rural
	views on vaccination outreach and delivery strategies.							
[33] [United States of America]	To explore where and how Black women are obtaining information that pertains to COVID-19, along with the impacts that COVID-19 may be having on their daily lives.	15	General public	Black/African American	NR	25.4<br>NR<br>(18-31)	All female	Urban
[34] [United States of America]	To determine (a) whether actual community-level risk influenced individual perceptions of community-level and personal risk and (b) how self-assessment of personal risk was reflected in the adoption of COVID-19 precautionary behaviours.	20	General public	Black	NR	46<br>NR<br>(18-55)	14 females (70%)<br>6 males (30%)	NR
[35] [Hong Kong]	To explore the lived experiences and challenges encountered by South and Southeast Asian women in Hong Kong during the COVID-19 pandemic.	10	South Asian and Southeast Asian women	Indian (3); Nepalese (3); Pakistani (2); Philippine (1); Indonesian (1)	NR	37<br>NR<br>(22-51)	All women	Urban
[36] [United Kingdom]	To develop action points and developing solutions to strengthen delivery and up- take of COVID-19 vaccine in marginalised migrant communities.	32	Migrants:<br>Asylum seekers (19); Undocumented (8) Refugees (3); Limited leave to remain (2)	African (11); Eastern Mediterranean (11); European (5); Sri Lankan (4); Venezuelan (1);	NR	37.1<br>NR<br>NR	21 females (66%)<br>11 males (34%)	NR
[37] [United Kingdom]	To gain an understanding of factors affecting hesitancy in Black and Asian individuals in England, to help address concerns about having the vaccine.	95	General public	Caribbean (25); Pakistani (24); African (22); Bangladeshi (16); Other (5); Mixed (3)	Muslim (44); Christian (37): No religion (10); Other (4)	NR<br>NR<br>(18-70+)	51 females (54%)<br>42 males (44%)<br>2 other (2%)	NR

Table S1. Included study aims and participant demographic characteristics (supplementary file).

Papers [Country]	Aims	Participant numbers	Participant type	Ethnicity and/or nationality	Religion	Age in years: Mean [Median] (Range)	Gender (%)	Urban/ rural
[38] [Finland]	To describe the lived experiences and meaning making of Russian-, Somali- and Arabic-speaking migrants in relation to COVID-19, and their everyday responses to the threat of the virus.	209	Migrants living in Finland	Arabic (Iraqi; Syrian; Palestinian - 65); Somali (76); Russian speaking (68)	NR	179 aged between 40 and 69. Others under 39 or over 70.	149 females (71%) 60 males (29%)	NR
[39] [United States of America]	To explore perceptions and lived experiences of African American pastors addressing the onslaught of COVID-19 with their congregation.	37	African American pastors	African American	NR	NR NR NR	NR	Urban and rural
[40] [Canada]	To explain how, for Chinese immigrants, international political tensions are implicated in a range of mental health–related phenomena.	25	Adult migrants from mainland China	Chinese	NR	NR NR (19+)	12 females (48%) 13 males (52%)	NR
[41] [United States of America]	To understand how both structural and SDOH shape perceptions of the coronavirus, its spread, and decision making around COVID-19 testing and vaccination in vulnerable populations.	53	Latinx and Indigenous Mexican immigrant communities	Latinx (44) and Purépecha (9)	NR	NR NR (18-64)	43 females (81%) 10 males (19%)	Rural
[42] [Norway]	To explore immigrants' perspectives of the factors that impede their adherence to preventive measures against Covid-19.	88	Immigrant ethnic groups	Somalia (12); Iraq (12); Pakistan (12); Afghanistan (11), Poland (11); Sri Lanka (6); Turkey (6); Bosnia/Serbia (6); Eritrea (6); Syria (6).	NR	NR NR (19-78)	49 females (56%) 39 males (44%)	NR
[43] [United Kingdom]	To address a lack of in-depth qualitative research exploring people with pre-existing mental health	49	People living with mental health conditions	White (31); Black (7); Asian (6); Mixed (4); Other (1)	NR	NR NR (18 to 70+)	34 females (69%) 15 males (31%)	Urban and rural

Table S1. Included study aims and participant demographic characteristics (supplementary file).

Papers [Country]	Aims	Participant numbers	Participant type	Ethnicity and/or nationality	Religion	Age in years: Mean [Median] (Range)	Gender (%)	Urban/ rural
	conditions experiences and perceptions of how life has changed due to COVID19.							
[44] [United States of America]	To explore the nature and impact of COVID-19 anti-Asian discrimination within a multi-racial sample.	95	Young adults	White (43); Asian (33); Black (7); Mixed (6); Latinx (4); Other (2)	NR	24.5<br>NR<br>(18-30)	81 females (85%)<br>10 males (11%)<br>4 non-binary (4%)	NR
[45] [United Kingdom]	To explore the perceptions of risk and experiences of social distancing and social isolation recommendations to reduce the risk of COVID-19 transmission, of Muslims living in the Northwest of England.	47	Muslim community	Multiple: British Arab/ British Yemeni/Arab; British Pakistani; Indian/Asian/ Bangladeshi/Pakistani; White European; Black British; Somali; Black African	Muslim	37.5<br>NR<br>(18-65)	25 females (53%)<br>22 males (47%)	NR
[46] [Sweden]	To understand this dilemma by investigating ethnic minorities' perceptions and their experience of accessing the COVID-19 vaccine.	25	Health volunteers (14), and people from minority groups (11)	Somali and Syrian (health volunteer data NR)	NR	NR<br>NR<br>(34–67)	6 females (55%)<br>5 males (45%)<br>(health volunteer data NR)	NR
[47] [United Kingdom]	To explore how the pandemic has impacted the identity of Staff of the Global Majority care home workers	14	Staff of the Global Majority care home workers	Black Caribbean (3); Black African (3) South Asian (3); Romani Gypsy (2); Arab (1); Austronesian (1)	NR	42<br>NR<br>(22-62)	10 females (71%)<br>4 males (29%)	NR
[48] [United Kingdom]	To explore the experiences of British Muslim Pakistani women with family responsibilities during the COVID-19	25	British Muslim Pakistani women	British Pakistani	Muslim	NR<br>NR<br>NR	All female	NR

Table S1. Included study aims and participant demographic characteristics (supplementary file).

Papers [Country]	Aims	Participant numbers	Participant type	Ethnicity and/or nationality	Religion	Age in years: Mean [Median] (Range)	Gender (%)	Urban/ rural
	pandemic, two years on from the first lockdown.							
[49] [United States of America]	To explore the experiences of Black and Latinx communities during the pandemic to better understand their perspectives on COVID-19 mitigation behaviours, testing and vaccines.	111	Black and Latinx communities in the USA	African American (68); Latinx (43)	NR	NR [43] (18-93)	87 females (78%) 24 males (22%)	NR
[50] [Spain]	Investigates the effect of lockdowns and pandemic restrictions on the home through the lens of Romanian Roma migrant women in Spain during the pandemic.	18	Roma migrant adult women in Spain	Roma ethnic Romanian	Christian Orthodox (15); Pentecostal (3)	NR NR (18-48)	All females	Urban
[51] [Canada]	To explore the perceptions of COVID-19 risk, vaccine access, uptake and confidence among South Asians living in Canada.	25	General public	South Asian	NR	NR [32] (19-69)	NR	NR
[52] [Canada]	To describe the nature of COVID-19 online disinformation among Black Canadians and identify the factors contributing to this phenomenon.	30	Community leaders	Black African	Christian (27); Muslim (2); Other (1)	NR NR (25-55+)	14 females (47%) 16 males (53%)	NR
[85] [United States of America]	To explore the attitudes of African Americans in New Orleans toward COVID-19, social and normative conditions which affected individual behaviours, as well as access to healthcare services and COVID-19 testing.	102	General public	African American	NR	40 NR (18-60+)	69 females (68%) 33 males (32%)	NR
[53]	To understand the pandemic's impact on recently arrived migrants and their	81	Migrants to the UK (15 asylum seekers and 2	Migrants: Ethnicity NR	NR	Migrants: 38	Migrants:	Urban and rural

Table S1. Included study aims and participant demographic characteristics (supplementary file).

Papers [Country]	Aims	Participant numbers	Participant type	Ethnicity and/or nationality	Religion	Age in years: Mean [Median] (Range)	Gender (%)	Urban/ rural
[United Kingdom]	access to primary health care, and implications for vaccine roll-out.	(17 recently arrived migrants and 64 primary care professionals - PCPs)	refugees) and primary care professionals (e.g., general practitioners and nurses)	PCPS: Ethnicity: African (4); Mixed (3); Caribbean (1); Indian (11); Pakistani (3); Asian Other (2); White British (32); White Irish (3) White Other (5)		NR (22-59) PCPs: 45 NR NR	11 females (65%) 6 males (35%) PCPs: 54 females (84%) 10 males (16%)	
[54] [United States of America]	To identify lay explanations for racial disparities in COVID-19 illness and death in St. Louis and examine whether and how they differ by a person's race, education, income, and age.	54	General public	Black (33); White (14); Other (7)	NR	46.8 NR (21-73)	44 females (81%) 10 males (19%)	NR
[55] [United Kingdom]	To understand how and why Gypsy, Roma and Traveller (GRT) groups in England decided to take up or to avoid COVID-19 vaccinations.	47	Individuals from GRT communities	Individuals from GRT communities	NR	NR [30] (18-50+)	33 females (70%) 14 males (30%)	Urban and rural
[56] [Canada]	Explores the impact of the pandemic on health services for First Nations people living with HIV (FN-PWLE) in Manitoba, Canada.	25 (n = 11 HCWs; n = 14 First Nations people living with HIV)	Healthcare providers (HCW) and First Nations people	Cree; Ojibwa; Dene; Oji-Cree; other (n = 14 FN-PWLE; n = 4 HCWs) Non-indigenous (n = 0 FN-PWLE; n- = 7 HCWs)	NR	FN-PWLE: NR [42.6] (25-59) HCW: NR [42.2] (30-60)	NR (numbers repressed due to confidentiality concerns)	Urban
[57] [Canada]	To examine how the COVID-19 pandemic has affected Asian	32	Asian Canadians	East Asian (14); South Asian (10); Southeast	NR	NR [35]	18 females (56%)	NR

Table S1. Included study aims and participant demographic characteristics (supplementary file).

Papers [Country]	Aims	Participant numbers	Participant type	Ethnicity and/or nationality	Religion	Age in years: Mean [Median] (Range)	Gender (%)	Urban/ rural
	Canadians' sense of safety and belonging in their Canadian communities.			Asian (5); West Asian (3)		NR	14 males (44%)	
[58] [United Kingdom]	Aimed to uncover the effect of the COVID-19 outbreak on young people with ethnic minority backgrounds' mental health.	10	Minority ethnic young people	Black or black African (4); black or black Caribbean (1); any other black background (1); mixed (3); prefer not to say (1)	NR	14.3<br>NR<br>(12-17)	7 females (70%)<br>3 males (40%)	Urban
[86] [United States of America]	To understand how COVID-19 affected Latinx sexual minority men (LSMM) and transgender women (LTGW).	52	Latinx sexual minority men and Latinx transgender women	Latinx	NR	39.9<br>NR<br>NR	16 transgender females (31%)<br>36 males (69%)	NR
[59] [United Kingdom]	To explore the perspectives of BAME community leaders in relation to the impact of the COVID-19 pandemic on their communities; and BAME community's perception, understanding and adherence to Government guidelines on COVID-19 public health measures.	19	BAME community leaders	NR – but various BAME ethnic community groups covered in the community leaders' roles	NR	NR<br>NR<br>NR	NR	NR
[60] [United States of America]	Explore vaccine attitudes and intentions among program participants, understand the role of an African American faith-based wellness program in COVID-19 awareness and vaccine uptake, and solicit potential solutions for this deep-rooted public health problem.	21	Church leaders, lifestyle coaches and programme participants	Black or African American (20); White (1)	Christian	62.8<br>NR<br>(30-60+)	15 Female (71%)<br>6 males (29%)	NR

Table S1. Included study aims and participant demographic characteristics (supplementary file).

Papers [Country]	Aims	Participant numbers	Participant type	Ethnicity and/or nationality	Religion	Age in years: Mean [Median] (Range)	Gender (%)	Urban/ rural
[87] [Albania]	To explore the accessibility of developmental assets among Egyptian and Roma minority youth in Albania during the COVID-19 pandemic.	31	Adolescents	Egyptian (16); Roma (15)	NR	16.7<br>NR<br>(14-20)	14 females (45%)<br>17 males (55%)	Urban
[61] [United States of America]	To understand COVID-19 vaccine decision-making among Black women in the United States.	60	Black women	African American/Black African (54); mixed (6)	NR	NR<br>NR<br>(19-75)	All females	Urban
[62] [United States of America]	To better understand perceptions of COVID-19 disease and prevention among unvaccinated Hispanic adults.	20	Hispanic adults	Hispanic	NR	NR<br>NR<br>NR	15 females (75%)<br>5 males (25%)	Urban
[63] [United States of America]	To assess and compare the self-reported impact and challenges caused by COVID-19 in Mexican-origin, Hispanic parents.	96	Mexican-origin parents and guardians of young adolescents	Hispanic	NR	39.8<br>NR<br>NR	92 females (96%)<br>4 males (4%)	NR
[64] [United States of America]	To understand the perception of the Latino community in a rural state regarding COVID-19.	14	Rural Latino community in the USA	Ethnicity: Latino<br>Nationality: Mexican (9); Colombia (2); Venezuela (2); Ecuador (1)	Catholic	43<br>NR<br>(30-59)	6 females (43%)<br>8 males (57%)	Rural
[65] [United Kingdom]	To explore the drivers for vaccine hesitancy in ethnic minority groups in the UK, the impact of social media on vaccine hesitancy and how vaccine hesitancy may be overcome.	12	Ethnic minority groups in the UK	Described as Black, Arab and Asian backgrounds	NR	NR<br>NR<br>NR	7 females (58%)<br>5 males (42%)	NR
[88] [United States of America]	To understand the barriers that Filipino care workers in the United States face in terms of workplace protection and safety during the COVID-19 pandemic.	89 (took part in the qualitative aspect: *kuwentuhan*)	Home care workers	Filipino/x	NR	NR<br>NR<br>(35-44)	NR	NR

Table S1. Included study aims and participant demographic characteristics (supplementary file).

Papers [Country]	Aims	Participant numbers	Participant type	Ethnicity and/or nationality	Religion	Age in years: Mean [Median] (Range)	Gender (%)	Urban/ rural
[66] [Canada]	To answer the following questions: As the COVID-19 pandemic took hold, was the intersecting relationship between social determinants of health embodied in public health and policy responses? What were the emerging impacts of COVID-19 on social and health inequities?	13	Indigenous elders and knowledge keepers, policy and decision makers, practitioners, and researchers from health and non-profit sectors	NR	NR	NR<br>NR<br>NR	NR	NR
[67] [Canada]	To explore contexts that influence COVID-19 vaccine uptake, barriers to vaccination, and vaccine hesitancy among predominantly racialized sexual and gender minority individuals.	40	Racialised sexual and gender minority individuals	Black (17); Asian (12); White (7); Latinx (3); mixed (1)	NR	29<br>NR<br>(19-58)	19 females (47.5%)<br>13 transgender (32.5%)<br>5 nonbinary (12.5%)<br>3 males (7.5%)	Urban
[68] [Belgium]	To explore information and prevention needs, coping mechanisms with COVID-19 control measures and their impact on lived experiences among selected racialized/ethnic minority communities.	71	Ethnic minorities in Belgium	African (37); Jewish communities (14); Belgian (12); Turkish (5); Syrian (2); Indian (1)	NR	NR<br>NR<br>NR	26 females (37%)<br>45 males (63%)	NR
[69] [United States of America]	To explore the experiences of Black carers of people living with dementia during the COVID-19 pandemic in the United States.	19	Informal carers of people living with dementia	All Black	NR	60<br>NR<br>(46-75)	17 females (89%)<br>2 males (11%)	NR
[70] [United Kingdom]	To draw on health and sociological theories of structure and agency to inform understanding of how	22	General public (14); Health professionals involved in COVID-19	Of members of the public:	NR	45.3<br>NR<br>(21-67)	General public:<br>9 females (64.3%)	Urban

Table S1. Included study aims and participant demographic characteristics (supplementary file).

Papers [Country]	Aims	Participant numbers	Participant type	Ethnicity and/or nationality	Religion	Age in years: Mean [Median] (Range)	Gender (%)	Urban/ rural
	structural factors influence vaccine confidence in low-uptake ethnic minority groups in London and the surrounding areas.		vaccination campaigns (8)	Bangladeshi (4); Caribbean (3); Pakistani (2); mixed (2); African (1); Indian (1); prefer not to say (1)			5 males (35.7%)<br><br>Health professionals: NR	
[71] [United States of America]	To qualitatively explore the impact of the COVID-19 pandemic within a racially/ ethnically diverse sample of GBMSM living with HIV.	15	Gay and bisexual men living with HIV	African American (6); Latinx (5); White (4)	NR	28<br>NR<br>NR	All males	NR
[72] [United States of America]	To explore experiences and adaptations during the COVID-19 pandemic.	18	Gay women	African American (9); Latinx (6); White (3);	NR	51.7<br>NR<br>(33-72)	All females	Urban
[89] [United States of America]	To examine: (1) adolescents' perceptions of how their social and emotional lives had changed during COVID-19; and (2) associations between these perceived changes and indices of their mental health.	407	Adolescents	White (212); African American (82); Latinx (67); Asian American (12); American Indian (4); Other/Mixed (29); missing (1)	NR	15.4<br>NR<br>(14-17)	203 females (49.9%)<br>204 males (50.1%)	Urban and rural
[73] [United Kingdom]	To explore the experiences, beliefs, feelings, and challenges faced by Pakistani migrant doctors working in the United Kingdom in times of the COVID-19 pandemic.	10	Medical doctors	Pakistani	NR	NR<br>NR<br>NR	6 males (60%)<br>4 females (40%)	NR
[74] [United States of America]	To focus on the qualitative experiences of Latinx nurses during the first wave of the pandemic.	20	Latinx nurses	Hispanic	NR	37.3<br>NR<br>(26-61)	18 females (90%)<br>2 males (10%)	NR

Table S1. Included study aims and participant demographic characteristics (supplementary file).

Papers [Country]	Aims	Participant numbers	Participant type	Ethnicity and/or nationality	Religion	Age in years: Mean [Median] (Range)	Gender (%)	Urban/ rural
[75] [United States of America]	To describe the cultural standpoint of 'aguantarismo', provide examples of its tactical deployment, and explores its role in undermining as well as supporting vaccine uptake.	42	Hispanic/Latino community	Hispanic/Latinx	NR	NR NR NR	28 females (67%) 14 males (33%)	NR
[76] [New Zealand]	To investigate the experiences and challenges of New Zealand registered nurses of Chinese ethnicity who have been working during the COVID-19 pandemic.	51	Nurses	Chinese	NR	NR NR (20-59)	49 females (96%) 2 males (4%)	NR
[90] [United States of America]	To explore the impact of the pandemic on the mental health and well-being of Latinx caregivers of children with intellectual and developmental disabilities.	37	Informal carers	Latinx	NR	44 NR NR	NR	Urban
[77] [United Arab Emirates]	To examine the experiences of twenty Black African social workers during the Coronavirus disease (COVID-19) pandemic in England.	20	Social workers	Black African	NR	NR NR NR	16 females (80%) 4 males (20%)	NR
[91] [United States of America]	To compare child and household experiences at the time of a child's COVID-19 diagnosis between immigrant and US-born parents and explore immigrant Latino perspectives on underlying causes of COVID-19 disparities between immigrant and US-born families.	16	Latino communities	Mexico (14); other non-US (2)	NR	42 NR NR	All females	NR
[78]	To understand how ethnic minority groups in the United Kingdom	57	General public	African (19); Indian (11); Caribbean (10);	NR	NR NR	37 females (65%)	NR

Table S1. Included study aims and participant demographic characteristics (supplementary file).

Papers [Country]	Aims	Participant numbers	Participant type	Ethnicity and/or nationality	Religion	Age in years: Mean [Median] (Range)	Gender (%)	Urban/ rural
[United Kingdom]	conceptualised COVID-19 and how this influenced engagement in testing.			Pakistani (9); Bangladeshi (8)		(18-61+)	20 males (35%)	
[79] [United States of America]	To examine factors influencing decisions to test for COVID-19 among Native Americans on the Flathead Reservation in Montana and the Latino community in the Yakima Valley of Washington state.	69	Community leaders and members	American Indian (29); Latino (31); White (15); Black (1); other (1)	NR	NR NR NR	52 females (75%) 17 males (25%)	Rural
[80] [United States of America]	To use COVID-19 vaccine trial participants' experiences to identify key themes in the lived experience of vaccination early in the vaccine approval and distribution process.	27	General public	White (20); Latinx (4); East Asian (2); Black (1)	NR	NR NR (18-64)	15 females (56%) 12 males (44%)	NR
[81] [United Kingdom]	To explore the effects of the COVID-19 pandemic on people living with dementia and their family carers of BAME backgrounds.	15	People living with dementia (4); and their informal carers (11)	Caribbean (7); Indian (7); Pakistani (1)	NR	NR NR (<50-80+)	12 females (80%) 3 males (20%)	NR
[82] [United Kingdom]	To better understand lower uptake among racial and ethnic minority staff groups to inform initiatives to enhance uptake.	25	Healthcare workers	White (14); Black (6); Asian (4); Other (1)	NR (only described as either religious or not religious)	NR NR NR	18 females (72%) 6 males (24%) 1 other (4%)	NR
[92] [United Kingdom]	To understand levels of vaccine hesitancy and the factors predicting this in UK HCWs using interim data from the United Kingdom	99	Healthcare workers	White (52); Asian (21); Black (16); Other (10)	NR	NR NR NR	75 females (76%) 24 males (24%)	NR

Table S1. Included study aims and participant demographic characteristics (supplementary file).

Papers [Country]	Aims	Participant numbers	Participant type	Ethnicity and/or nationality	Religion	Age in years: Mean [Median] (Range)	Gender (%)	Urban/ rural
	research study into ethnicity and COVID-19 outcomes in healthcare workers.							

Note: NR = not reported; NA = not applicable; WHO = World Health Organisation; BAME = Black Asian and Minority Ethnic; HCWs = healthcare workers; GBMSM = gay, bisexual, and other men who have sex with men.
