# Supplementary material for: The Impact of COVID-19 on Racialised Minority Populations: A Systematic Review of Experiences and Perspectives
Source: Int J Environ Res Public Health. 2025 Nov 21;22(12):1767. doi: 10.3390/ijerph22121767 (PMC12732843; doi:10.3390/ijerph22121767)
Supplement: Supplementary file 1 [file ijerph-22-01767-s001.zip › ijerph-3809498 - table S2.pdf]

Table S2. Included study methods and quality scores (supplementary file).

Papers [Country]	Study type	Design	Sampling	Data collection	Data analysis	Quality scores
[8] [United States of America]	Qualitative	Cross-sectional	Purposive	One-to-one, telephone, semi structured interviews	Coding based on grounded theory and thematic analysis	80%
[9] [United States of America]	Qualitative	Cross-sectional	Purposive	Online virtual focus groups	Thematic analysis	100%
[12] [United States of America]	Qualitative	Cross-sectional	Purposive and snowball	One-to-one, semi-structured, telephone interviews	Thematic analysis	100%
[13] [United States of America]	Qualitative	Cross-sectional	Purposive	One-to-one, semi-structured, telephone interviews	Thematic analysis	100%
[27] [Spain]	Qualitative	Cross-sectional	Purposive	Conference/focus group	Thematic analysis	100%
[28] [Denmark]	Qualitative	Cross-sectional	Snowball	One-to-one in-person interviews	Thematic analysis	100%
[29] [United States of America]	Qualitative	Cross-sectional	Convenience	Focus groups	Thematic content analysis	100%
[30] [United States of America]	Qualitative	Cross-sectional	Purposive	Virtual focus groups	Thematic analysis	100%
[83] [United Kingdom]	Mixed methods	Cross-sectional	Purposive	One-to-one, semi-structured virtual online interviews	Thematic analysis	80%
[84] [United States of America]	Multiple methods	Cross-sectional	Convenience	Online survey	Coding	60%
[31] [Chile]	Qualitative	Cross-sectional	Purposive	Online virtual one-to-one semi-structured interviews	Thematic content analysis	100%
[32] [United States of America]	Qualitative	Cross-sectional	Convenience	Face-to-face, one-to-one, focus groups and semi-structured interviews	Modified grounded theory	80%
[33] [United States of America]	Qualitative	Cross-sectional	Purposive	Face-to-face, one-to-one, semi-structured interviews	Thematic analysis	100%
[34] [United States of America]	Qualitative	Cross-sectional	Purposive	One-to-one online semi-structured interviews	Constant comparative analysis	100%
[35]	Qualitative	Cross-sectional	Purposive	Face-to-face semi-structured interviews	Thematic analysis	100%

Table S2. Included study methods and quality scores (supplementary file).

Papers [Country]	Study type	Design	Sampling	Data collection	Data analysis	Quality scores
[Hong Kong]						
[36] [United Kingdom]	Qualitative	Cross-sectional	Purposive and snowball	One-to-one, telephone, semi structured interviews	Interpretative approach through a thematic framework	100%
[37] [United Kingdom]	Qualitative	Cross-sectional	Convenience and snowball	Online focus group workshops	Inductive thematic analysis	60%
[38] [Finland]	Qualitative	Cross-sectional	Snowball	One-to-one, telephone, semi structured interviews	Thematic analysis	100%
[39] [United States of America]	Qualitative	Cross-sectional	Purposive and snowball	Face-to-face, one-to-one, semi-structured interviews	Framework analysis	100%
[40] [Canada]	Qualitative	Cross-sectional	Convenience and snowball	One-to-one, telephone, semi structured interviews	Discourse analysis	60%
[41] [United States of America]	Qualitative	Cross-sectional	Snowball	Online virtual focus groups	Described as a qualitative analytic technique	100%
[42] [Norway]	Qualitative	Cross-sectional	Purposive and snowball	One-to-one, semi-structured interviews	Thematic analysis	100%
[43] [United Kingdom]	Qualitative	Cross-sectional	Purposive	One-to-one, virtual online, semi structured interviews	Thematic analysis	100%
[44] [United States of America]	Qualitative	Cross-sectional	Convenience and snowball	Online survey	Thematic analysis	60%
[45] [United Kingdom]	Qualitative	Cross-sectional	Purposive	One-to-one, semi-structured interviews and focus groups	Thematic analysis	100%
[46] [Sweden]	Qualitative	Cross-sectional	Purposive	One-to-one, virtual online, interviews	Deductive qualitative analysis	100%
[47] [United Kingdom]	Qualitative	Cross-sectional	Convenience and theoretical	Online or telephone one-to-one, semi-structured interviews	Constructivist grounded theory	100%
[48] [United Kingdom]	Qualitative	Cross-sectional	Purposive	One-to-one, telephone, semi structured interviews	Coding	100%
[49] [United States of America]	Qualitative	Cross-sectional	Purposive	Online virtual focus group and individual interviews	Described as an editing approach	100%

Table S2. Included study methods and quality scores (supplementary file).

Papers [Country]	Study type	Design	Sampling	Data collection	Data analysis	Quality scores
[50] [Spain]	Qualitative	Cross-sectional	Purposive	Focus group interviews	Thematic analysis	100%
[51] [Canada]	Qualitative	Cross-sectional	Convenience	Online one-to-one, semi-structured interviews	Thematic analysis	100%
[52] [Canada]	Qualitative	Cross-sectional	Purposive and snowball	One-to-one in-depth interviews	Content analysis	60%
[85] [United States of America]	Multiple methods	Cross-sectional	Purposive	Online survey	Content analysis	80%
[53] [United Kingdom]	Qualitative	Cross-sectional	Purposive; convenience and snowball	One-to-one, telephone, semi structured interviews	Thematic analysis	100%
[54] [United States of America]	Qualitative	Cross-sectional	Convenience	Online virtual individual interviews	Directed content analysis	100%
[55] [United Kingdom]	Qualitative	Cross-sectional	Purposive	In-person and online dialogue sessions, Interviews	Iterative methods of constant comparison	100%
[56] [Canada]	Qualitative	Cross-sectional	Purposive; snowball	Online interviews	Inductive content analysis	100%
[57] [Canada]	Qualitative	Cross-sectional	Purposive and snowball	One-to-one, telephone or online, semi structured interviews	Thematic analysis	60%
[58] [United Kingdom]	Qualitative	Cross-sectional	Convenience	One-to-one semi structured interviews	Interpretative phenomenological analysis	100%
[86] [United States of America]	Mixed methods	Cross-sectional	Random (from a larger study)	One-to-one, telephone, semi structured interviews	Directed content analysis	100%
[59] [United Kingdom]	Qualitative	Cross-sectional	Purposive and snowball	One-to-one, telephone or online, semi structured interviews	Thematic analysis	100%
[60] [United States of America]	Qualitative	Cross-sectional	Purposive and snowball	Telephone and in-person interviews	Inductive and deductive hematic analysis	100%
[87] [Albania]	Mixed methods	Cross-sectional	Convenience	Focus groups	Thematic analysis	100%

Table S2. Included study methods and quality scores (supplementary file).

Papers [Country]	Study type	Design	Sampling	Data collection	Data analysis	Quality scores
[61] [United States of America]	Qualitative	Cross-sectional	Social media purposive and snowball	In-depth semi structured online interviews	Thematic analysis	80%
[62] (2023) [United States of America]	Qualitative	Cross-sectional	Social media and web-based purposive	Online and in-person interviews	Conventional content analysis	100%
[63] (2022) [United States of America]	Qualitative	Cross-sectional	Purposive	Survey and telephone, structured interviews	Described as a qualitative data coding process	40%
[64] [United States of America]	Qualitative	Cross-sectional	Snowball	One-to-one, telephone, semi structured interviews	Thematic analysis using the framework method	100%
[65] [United Kingdom]	Qualitative	Cross-sectional	Snowball	One-to-one, virtual online, semi structured interviews	Thematic analysis	80%
[88] [United States of America]	Mixed methods	Cross-sectional	Convenience	Semi-structured interview; 'kuwentuhan' (story-telling)	Collaborative data analysis; racial capitalism analysis	80%
[66] [Canada]	Qualitative	Cross-sectional	Convenience	Online virtual facilitated conversations	Thematic analysis	80%
[67] [Canada]	Qualitative	Cross-sectional	Purposive	Online in-depth semi structured interviews	Interpretive thematic analysis	100%
[68] [Belgium]	Qualitative	Cross-sectional	Convenience and snowball	Online virtual group discussions and individual in-depth interviews	Reflexive thematic approach	100%
[69] [United States of America]	Qualitative	Cross-sectional	Purposive; convenience and snowball	Online virtual focus groups	Thematic analysis	100%
[70] [United Kingdom]	Qualitative	Cross-sectional	Purposive and snowball	Online one-to-one interviews and focus groups	Inductive reflective thematic analysis	100%
[71] [United States of America]	Qualitative	Cross-sectional	Purposive; convenience and snowball	One-to-one, telephone, semi structured interviews	Constant comparison analysis	100%

Table S2. Included study methods and quality scores (supplementary file).

Papers [Country]	Study type	Design	Sampling	Data collection	Data analysis	Quality scores
[72] [United States of America]	Qualitative	Cross-sectional	Purposive	One-to-one, telephone or virtual online, semi structured interviews	Phenomenological analysis	100%
[89] [United States of America]	Mixed methods	Longitudinal	Stratified random	Open-ended survey responses	Thematic analysis	60%
[73] [United Kingdom]	Qualitative	Cross-sectional, phenomenological approach	Purposive and snowball	One-to-one, semi-structured telephone interviews	Described as an inductive qualitative approach	100%
[74] [United States of America]	Qualitative	Cross-sectional	Purposive	Telephone interviews	Descriptive thematic analysis	100%
[75] [United States of America]	Qualitative	Cross-sectional	Convenience	Ethnographically informed one-to-one, semi-structured telephone; virtual online and in-person interviews	Coding	80%
[76] [New Zealand]	Qualitative	Cross-sectional	Convenience	Online questionnaire/survey	Thematic analysis	100%
[90] [United States of America]	Mixed methods	Cross-sectional	Purposive	One-to-one, semi-structured telephone and virtual online interviews	Described as a team-based analytic approach	100%
[77] [United Arab Emirates]	Qualitative	Cross-sectional	Convenience	One-to-one, semi-structured virtual online interviews	Thematic analysis	80%
[91] [United Stated of America]	Mixed methods	Cross-sectional	Purposive	Focus groups	Coding	80%
[78] [United Kingdom]	Qualitative	Cross-sectional	Purposive	One-to-one, semi-structured telephone and virtual online interviews	Grounded theory	100%
[79] [United States of America]	Qualitative	Cross-sectional	Purposive	Interviews and focus groups	Deductive coding and iterative thematic analysis	100%
[80] [United States of America]	Qualitative	Cross-sectional	Convenience	One-to-one, semi-structured telephone interviews	Thematic analysis	100%
[81] [United Kingdom]	Qualitative	Cross-sectional	Purposive	One-to-one, semi-structured telephone and virtual online interviews	Thematic analysis	100%

Table S2. Included study methods and quality scores (supplementary file).

Papers [Country]	Study type	Design	Sampling	Data collection	Data analysis	Quality scores
[82] [United Kingdom]	Qualitative	Cross-sectional	Snowball and key informant sampling	One-to-one, semi-structured interviews	Thematic analysis	100%
[92] [United Kingdom]	Mixed methods	Prospective longitudinal cohort and qualitative study	Purposive	Semi-structured interviews and focus groups	Framework analysis	60%

Note: NR = not reported; NA = not applicable
