# Supplementary material for: The Impact of COVID-19 on Racialised Minority Populations: A Systematic Review of Experiences and Perspectives
Source: Int J Environ Res Public Health. 2025 Nov 21;22(12):1767. doi: 10.3390/ijerph22121767 (PMC12732843; doi:10.3390/ijerph22121767)
Supplement: Supplementary file 1 [file ijerph-22-01767-s001.zip › ijerph-3809498 - table S3.pdf]

Table S3. Indicative quotations (supplementary file).

Theme	Indicative quotations
Children and young people	"Before this we didn't really know our kids. The kids didn't really know us...so I think that's the opportunity is to connect at the deeper level" (African American) [13]<br><br>"The situation became very difficult during the pandemic because my father had no job, my mother was sick, and we had no money to buy medicine or bread. We were very scared as we did not know how long this situation would last" (Egyptian and Roma Albanian) [87]<br><br>"Not being able to play in my basketball team, this makes me really angry, sad, and depressed" (African American; Latinx; Asian American; American Indian; Other/Mixed) [89]<br><br>". . .my daughter was receiving. . .services before for mental health and now, they are cancelled. I am worried about the limited social interactions the girls [child with a disability and her sibling] are having and it has had a negative impact on their progress" (Latinx) [90]
Exacerbated pre-existing disparities	"They don't need to put the word documents [in Covid vaccine adverts] because.. what if I don't have it, I'm undocumented. And you said okay come on have your vaccine, we're not going to check you.. I won't go because I don't know to what extent is true. It might be a ploy to get people to come" (African; Eastern Mediterranean; Sri Lankan; Venezuelan) [36]<br><br>"There is fear that you can lose your job if you test positive for the virus." (Latinx and Purépecha) [41]<br><br>"Many immigrants live in overcrowded living conditions. If a member in the family gets infected, the other members in the family have no chance to protect themselves from the virus. I know an Afghan family whose daughter got the infection at work...The whole family became infected" (Somalia; Iraq; Pakistan; Afghanistan, Sri Lanka; Turkey; Eritrea; Syria) [42]<br><br>"Some people only had access to computers... if they came to work and they haven't got computers at home... A lot of the older generation or people on low incomes, they've got the old-fashioned phones so they can't go on... things like Facebook and WhatsApp and…other digital platforms..." (BAME) [59]<br><br>"Indigenous peoples statistically have high rates of respiratory and heart disease, due to a variety of longstanding determinants of health stemming from continued colonization. These conditions make a person especially vulnerable to COVID-19" (Canadian Indigenous) [66]<br><br>"Many people from our community, about 80%, are disadvantaged people. Sitting with three, four children in an apartment without a garden ... for them it has actually been enough. Especially in big cities.. This has a lot to do with their social situation: housing, income, problematic family situations, problems, debts,... people have other

Table S3. Indicative quotations (supplementary file)**.**

Theme	Indicative quotations
	priorities: finding food for their children, for example, everything else takes a back seat" (Sub-Saharan Africa; North-African; Middle Eastern; Orthodox Jewish) [68]<br><br>"We knew that we were working hard at the butt end of racism. We knew that it is members of our community who were supporting the NHS, keeping the carer infrastructure alive in this country. We knew all of these things about ourselves. What happened with COVID is it forced other people who didn't see us, to see us, all of a sudden." "I think minorities are the ones that are working in the frontlines or doing the jobs that people don't want to do. Then I guess I'm more at risk in a sense." "I think somehow there's always this narrative that we're the other. COVID, obviously there's research and statistics showing that our communities have been affected far more greater than white counterparts. And so, that's another problem to us, if that makes sense …" "It becomes a challenge for those who work outside their home to be able to do it and have an income. Most immigrant families aren't that privileged. Most of us are in dirty, demeaning or deadly jobs." "People are worried not just about Covid-19 they are worried about their last meal. Some people will not want to say they have Covid-19 because they have to isolate. That means less income" (African; Indian; Caribbean; Pakistani; Bangladeshi) [78]<br><br>"It becomes a challenge for those who work outside their home to be able to do it and have an income. Most immigrant families aren't that privileged. Most of us are in dirty, demeaning or deadly jobs" (African; Indian; Caribbean; Pakistani; Bangladeshi) [78]
lack of knowledge and information about COVID-19 and COVID-19 misinformation	"The elderly people around me have not gotten vaccinated because the sites of vaccination are too far away". "We don't read English, and we don't know how to use the Internet well, so it's difficult" (Latinx; Black/African Americans; Chinese Americans) [32]<br><br>"Yes [the information about COVID-19] it's confusing because I have so many different stories about it [COVID-19] I don't know what to really believe". "I feel like the news doesn't report everything...they always spin it" (Black/African American) [33]<br><br>"For me, I would like to take the vaccine if that will make everything better. But the fake news is scaring me, so I don't know. That is a problem. I don't know if it's real, I don't know if it's fake. When you take it, it will change the DNA... it will stop the person not having kids in future. A lot of stories are flying" (African; Eastern Mediterranean; European; Sri Lankan; Venezuelan) [36]<br><br>"Perhaps they [community members] are confused because they see the Donald Trump administration, and he said, 'No, no, no.' . . . I think people are confused about believing whether the virus is serious or not" (Latinx and Purépecha) [41]

Table S3. Indicative quotations (supplementary file).

Theme	Indicative quotations
	"Some of the people, culturally, they don't believe that such a virus exists. They think that it's 5G or something else". "If I go there [for a vaccine] they might be using me as a guinea pig or I don't know. They might be using me for their own things. I don't trust them" (region of origin: Africa; South America; Eastern Mediterranean; Southeast Asian) [53]<br><br>"I think it is very confusing. The things they have on official websites, social media talks about it, and you don't know what to trust." (Black, Arab and Asian) [65]
Intercommunity mutual aid	"I truly believe that Roma women can do whatever we want, because during the lockdown, many Roma women created ourselves a food bank. The work we have done is huge, and many other Roma and non-Roma organizations have joined us in our effort to guarantee basic needs for Roma families. We helped almost 350 Roma families, so definitely we can achieve whatever we propose to do" (Roma) [27]<br><br>"This school year has been complicated because of COVID-19. So I created a study group with the organizations here in the neighborhood to seek help for academic support. Since I have ended the school year with good grades, I really think that we should create a similar study group like the one I had, but with Roma girls so we can support each other and keep our motivation through the academic journey." (Roma) [27]<br><br>"We have, as a Somali community, determined together to assemble the folks and communicate to our community the best and most reliable information" (Somali) [38]<br><br>"The food bank over the past six months has... been inundated with people from... the community... We started off a drop-off service as well during the first lockdown... We also have a counselling service as a listening service..., people, they just, they wanna [want to] talk" (BAME) [59]<br><br>"Our community has really stepped up to help people who are struggling, and we have benefitted from that as well. We have received food at no cost, tests at no cost, and other things like clothes, books, Christmas gifts" (Latinx) [90]
Racial history of medicine and treatment of racialised populations	"To be very honest—and it's getting back to a whole lot of things that have happened to our people back in the day. They don't trust doctors. They don't trust people...I haven't taken the test. I don't know if I'll take the test" (African American) [30]<br><br>"We feel that for decades, we have been on the receiving end of health and social inequalities. Now, we are being presented with a new vaccine that has been developed in quick time and we are now priority group number 1" (Asian/Asian British; Black/Black British; White Other; Mixed) [83]

Table S3. Indicative quotations (supplementary file).

Theme	Indicative quotations
	"This mistrust we have in all the systems, all the powers that be, they haven't historically treated Black people well in any system, whether it be medical, governmental, anything, so why start now?." "The Latinx community, the majority have a lot of fear in what they say: They deny us everything, right? We don't have health insurance; we don't have funds for anything; we don't count for anything because we are illegal, because we are immigrants, or whatever it maybe. Why now are they offering us this? Like, it's too good to be true, they want to use us" (Latinx; Black/African Americans; Chinese Americans) [32]<br><br>"My neighbors say 'No' [to getting the vaccine], because they [the government] are going to put a chip in them, or because they might put another virus [in them]... that's what people from my community think." (Latinx and Purépecha) [41]<br><br>"There are some people who think they would actually deliberately kill you if you go to hospital ... You aren't prioritised. They would just let you die" (African; Indian; Caribbean; Pakistani; Bangladeshi) [78]<br><br>"This tracing is it just people in general rather than with Covid. It's all control, really." "It was literally introduced in the evening before Eid and everybody was feeling really like, they've just done this because it's Eid tomorrow and they don't want us to mix. And then the policing, I think they've always felt overly policed, as it is. And now it's like, ethnic minorities have bigger families and they're more likely to be breaking the rules. And people are getting reported and I think they feel overpoliced in that regard" (African; Indian; Caribbean; Pakistani; Bangladeshi) [78]<br><br>"Most of the information [from healthcare experts] I trust, but who knows what to believe" (African American) [85]<br><br>"[Even if] they [people from the community] couldn't breathe […] they were very reluctant to get to go to hospital. […] They're just, within a lot of hospital settings, obviously, I'm not speaking about doctors because they're [there] to save lives but there's a lot of discrimination, you know what I mean" (GRT) [55]<br><br>"BME staff have been less likely to opt into receiving the vaccine [...] I think there's something around how...If you look at things historically, Black and Asian communities have been misused in research [...] we have been abused and violated in previous vaccination trials and we can't deny that." "I'm not sure if they've done any particular work with the BME staff network to understand why people might be apprehensive about the vaccine. There's not been any direct kind of involvement, or them reaching out to understand what the issue is" (Black; Asian) [82]<br><br>"As a […] patient, some of the drugs that we have as Black women, actually it's having a different effect on us…So I just wanted to know how many people from the Black community, the Asian community, had been involved in the

Table S3. Indicative quotations (supplementary file).

Theme	Indicative quotations
	trials." "I'm considering just doing my vaccine publicly and getting it out in the hospital media and the local media. Not just because I'm Black, you know, there are low-income households saying the same thing" (Asian; Black) [92]
Contemporary experiences of racism	"Trump saying that it's the China virus — it's obviously we cover so much US politics in Canada that I think it's influenced the crazy people in our country as well. Or it's resonated with them, I guess." "I eat more when depressed, leading to weight gain, and lack of vitamin D due to staying indoors for too long, and then stress and anxiety that's just always present compared to before the pandemic. It's just that the mental impacts of it can be sometimes really overwhelming." "All of the Asian hate crimes are scary, in my opinion. I am less willing to do things alone and go out into neighborhoods by myself"(East Asian; South Asian; Southeast Asian; West Asian) [57]<br><br>"With the news that people from Black and ethnic minority communities are more likely to get it, and die from it ... a fear of a lot of people has been that we'll go into hospital, and they'll look at me and say 'you're not really worth that much, and we just won't give you a ventilator if we don't have enough." "People have been rude and nasty, they think they will catch it if they stand next to you ... I was already in that margin of people who were stigmatised, and now we are all stigmatised." "They didn't have cultural training, wasn't their fault, but it felt alien to me ... we live in a multi-cultural Britain ...there should be more representation in services" (Black; Asian; Mixed) [43]<br><br>"It was scary to hear about my acquaintance getting screamed at and physically assaulted (pushed) in the town where we grew up. I never experienced significant discrimination growing up so this seems so far out of the ordinary." "I haven't faced discrimination, but I also haven't left the house closed to for 4 weeks.... In fact, I fear leaving the house because I have heard reports of multiple attacks in and around my neighborhood." "It is exhausting and almost makes me feel hopeless. Even though racism is being exposed in institutions, public figures, and authority, there is denial and resistance to reform them which makes me feel distressed about it" (Asian; Black; Mixed; Latinx) [44]<br><br>"...this year, 2020, obviously is like the perfect storm of everything going wrong. We have the politics, and the pandemic, and the weather, and God knows what else, and the riots." "It was scary and I was nervous. I was really anxious [about food access] and really motivated to start planting a garden indoors just in case we don't have access to food. Now I feel a little bit more prepared." "I don't want to go to any place where I can meet somebody. I don't want to be with nobody. I feel the pandemic has had an influence on that." "people are getting beat up all the time." "Most people I am seeing here are wearing masks [during protests]. While I understand and support the marches, I don't want to be in the wrong place at the wrong time. Whether it's because of a march or the looting or just the violent police nature, those are the things that make me stay more at home." "I guess now [racism] is noticeable to them. Now that microaggression is an everyday word, people are actually noticing. Black Lives Matter and what happened to George Floyd unfortunately crystallized exactly what people have been talking about for years about the racial injustice of some police officers" (African American; Latinx) [72]

Table S3. Indicative quotations (supplementary file).

Theme	Indicative quotations
	"(Anonymised person) I know your parents are in [Country name] in Africa, so you won't have grounds for shielding." "You can tell the unease arising from BLM for some of our white colleagues. I became more vocal and took part in the marches and so on. I would mention this during virtual meetings and no one would comment, let alone ask questions. For me, this is the time for us to have our voices heard." "Risk assessments introduced for Black staff were experienced as a tick box exercise and not as a protective mechanism for those at higher risk of COVID-19. "my risk assessment was done virtually. My manager asked if I felt well and healthy, whether I had (you know) any existing health conditions, I said no and that was it." "White service users were observed as being more demanding than Black service users and this meant they were given equipment that helped them access virtual services. "When we moved to virtual visits and phone calls, they accepted the new terms without question. Our White service users on the other hand demanded phones, internet credit, you know, and they were given these" (Black African) [77]<br><br>"...my patients accused me being a Chinese as the cause of the pandemic." (Chinese) [76]
Adherence to preventative guidance/COVID-19 restrictions	"In the Eastern culture, social contact is a very important aspect of our daily routine. I can't spend a day without meeting with my social network. In my view, I would have preferred to get the coronavirus infection rather than being isolated" (Somalia; Iraq; Pakistan; Afghanistan, Poland; Sri Lanka; Turkey; Bosnia/Serbia; Eritrea; Syria) [42]<br><br>"I think there's socioeconomic background of a lot of BAME communities is another factor and the fact say from a Muslim perspective we are very sociable as a community or communities, whether you're sort of Arab, Asian, African you tend to have back-grounds of living if not with family having a lot of involvement with your family even day to day interactions... so I think there's lots of that physical contact is very much part of it hugging and shaking hands and so I think there's a combination of things that probably makes us more at risk" (Arab; British Yemeni/Arab; British Pakistani; Indian/Asian/ Bangladeshi/Pakistani; Black British; Somali; Black African) [45]<br><br>"It came to light that ministers were actually breaking those guidelines". "People then thought it was a two-tier system... one rule for the ministers and one rule for... everybody else" (BAME) [59]<br><br>"I have been extremely social distancing. I, in fact, haven't left my house; some of my friends do the grocery shopping for me and leave my groceries by the door. I bring them inside, sanitize them, and throw the bags out" (African American; Latinx) [71]
Impact on physical and mental health and wellbeing<br><br>Multilocal concerns – moved to mental wellbeing	"Oh anxiety yes...[I have been getting] less sleep. I don't know why it's been harder lately to go to sleep" (Black/African American) [33]

Table S3. Indicative quotations (supplementary file)**.**

Theme	Indicative quotations
	"Everything is available, but my mental health is in a very poor state. The last time I experienced similar anxiety was in 1991 in Iraq during the war." "I can't say where I can get help. If I got a service in my own language, then I might know who to contact. [. . .] News [readers] speak quickly. Difficult to understand them. Finnish is the most difficult language" (Arabic; Iraqi; Syrian; Palestinian; Somali) [38]<br><br>"I have had at least two emotional breakdowns. It takes a lot to remove the focus off the crisis and refocus on other things" (African American) [85]<br><br>"It's [the pandemic and lockdown] just making the situation for people worse, in a way, because people will start having suicidal thoughts, starting to think about the country that you came here from, there's war, there's poverty" (region of origin: Africa; South America; Eastern Mediterranean; Southeast Asian) [53]<br><br>"But now what they've done, there's no way could you get that lock off. They've fitted a box round it, so you can't get into it. […] you'd have a heart attack and you'd be dead." "We had to have a gate codes broke off, because they refused to give us the code." "When there was a case of Covid on our site the postmen were told by our own wardens […] to not deliver letters." I feel like she was forcing me into it. […] it was like, oh I can book you in now, and I said, no, and she was like, oh, well, call me and I'll book you in then, I'll be there, and I said, no, and like I told her why [concerns about fertility] and she was like, no, that's not true, and everything." "they are bullying people to get it done […] if you don't have it, you don't have no freedom" (GRT) [55]<br><br>"What is going on with COVID there are times that this produces, anxiety, concern, it causes low moods." "I had approval to do the facial feminization and hair removal (face, mustache and beard, intimate parts), my appointments have been delayed and they don't know when I will get another appointment, until things are more calm with coronavirus… sometimes, so I can't see my doctor to see my levels of hormones, and they can't see me because they are not doing check-ups ... they can't check my blood well because they are not making lab [appointments] because they aren't receiving patients –it has affected my health in that way..." (Latinx) [86]<br><br>" ... the lockdown itself is very bad for people's physical and mental well-being (...) There are also a lot of separations. I've had to do three appointments this week for a divorce, ..., it's been difficult for everyone." "...measures were policed differently and that is not quite right. I observed this, I went to the square X [in a middle-class neighborhood], there are many outdoor pubs and benches, I see a slightly whiter target group. Ten go to square Y, [neighborhood with high proportion of foreign-born population)]. You see people coming outside as well, they are different. A lot more young people and families with migrant background, but there are a lot more fines issued there than in the other neighborhood. That's not the only incident, those are stories from youth

Table S3. Indicative quotations (supplementary file)**.**

Theme	Indicative quotations
	workers who have seen that for themselves" (Sub-Saharan Africa; North-African; Middle Eastern; Orthodox Jewish) [68]<br><br>"I think . . . it was the downfall of her . . . . I saw how she declined through this whole crisis. I think a lot of it was because she could not interact with anyone other than myself and her caregiver . . . I'm sure she was not alone in this." "With the pandemic, everything had to be on Zoom, and so it got a lot more stressful . . . you have a system in place and it just gets wrecked." "I think my husband just needed to be in the doctors' office, quite frankly." "I went to take the first one, and they had indicated there would be a line, and I was thinking about my husband. And I got there and I said, *There's no way he's gonna stand in this line and no way he's gonna keep the mask on*. So, I held back from getting it" (Black) [69]<br><br>"I am a single mother, I don't [have] a sitter. Mentally it [the pandemic] has also affected me. I had three panic attacks. We went through the virus, I felt it more" (Latinx) [90]<br><br>"We knew that we were working hard at the butt end of racism. We knew that it is members of our community who were supporting the NHS, keeping the carer infrastructure alive in this country. We knew all of these things about ourselves. What happened with COVID is it forced other people who didn't see us, to see us, all of a sudden." "I think minorities are the ones that are working in the frontlines or doing the jobs that people don't want to do. Then I guess I'm more at risk in a sense." "I think somehow there's always this narrative that we're the other. COVID, obviously there's research and statistics showing that our communities have been affected far more greater than white counterparts. And so, that's another problem to us, if that makes sense …" "It becomes a challenge for those who work outside their home to be able to do it and have an income. Most immigrant families aren't that privileged. Most of us are in dirty, demeaning or deadly jobs." "People are worried not just about Covid-19 they are worried about their last meal. Some people will not want to say they have Covid-19 because they have to isolate. That means less income" (African; Indian; Caribbean; Pakistani; Bangladeshi) [78]<br><br>Multilocal concerns<br>"I'm worried about my homeland. I am worried about both [countries]. We live here, and my family lives in [my] homeland" (Arabic; Iraqi; Syrian; Palestinian; Somali) [38]<br><br>"[I] have turned fragile, hypersensitive, and agitated. Go to work, get off from work, sleep, check my cellphone nonstop, suddenly wake up at midnight, check messages, chat with my (Chinese) classmates and friends, and go back to sleep again. Anxiety, hope, disappointment, worry, sadness, crying out loudly and uncontrollably; I have never experienced anything like this in my lifetime" (Chinese) [40]

Table S3. Indicative quotations (supplementary file).

Theme	Indicative quotations
	"People in Pakistan still think it's a conspiracy and there is no real such disease" (Pakistani) [73]
Concerns about safety at work	"I think I got COVID-19 because I was using public transportation to get to work ... when I would get on the bus, there were lots of people without face masks or gloves and it didn't look like they had their own hand sanitizer either." (Latinx) [8]<br><br>"When this COVID came, we were just told to use the . . . well, they provided us with PPE, but only very minimal…." (Filipino/x) [88]<br><br>"Despite all the PPEs, the fear of being affected and the risk factor is constantly with us. You don't feel safe there at all". "I can tell you I was one of the doctors who treated corona patients without any PPE" (Pakistani) [73]<br><br>"...most colleagues do not wear masks, and I give in to peer pressure." "I use my own PPE every day'. 'I isolate myself from other family members to protect them" (Chinese) [76]<br><br>"It was a big mistake. I was expected to come into the office, go on home visits and cover the cases of so many colleagues who were not working. I had taken annual leave just before the lockdown so I would be unpaid if I didn't continue to work." "Those of us not shielding became emotionally and physically exhausted. Most people who were in the offices were Black." "Yes, we were at risk of the covid virus itself, but we were also at risk from the people who made decisions about us as Black staff." "They [*managers*] would pretend to be interested in my health and wellbeing, just checking on me they would say…but would end each phone call with 'when are you returning to work?'" (Black African) [77]
The role of faith	"I just felt like God is in control. When one is a believer, one believes that everything is in God's hands. If I came back to my family or I died, it would be God's will" (Latinx) [8]<br><br>"I do my best, but God protects" (Arabic; Iraqi; Syrian; Palestinian; Somali) [38]<br><br>"I reminded myself that only what Allah wants will happen". "I was crying on the daily basis, patients were slipping from my hands [dying], the only console I had was to turning to God and seek help for my inner peace" (Pakistani) [73]<br><br>"Coronavirus is punishment from God for disobedience" (African; Indian; Caribbean; Pakistani; Bangladeshi) [78]
